# Virome analysis provides new insights into the pathogenesis mechanism and treatment of SLE disease

**DOI:** 10.3389/fcimb.2024.1484529

**Published:** 2024-10-24

**Authors:** Yifan Wu, Zhiyuan Zhang, Xinglian Wang, Xun Liu, Ye Qiu, Xingyi Ge, Zhichao Miao, Xiangxian Meng, Yousong Peng

**Affiliations:** ^1^ Hunan Provincial Key Laboratory of Medical Virology, College of Biology, Hunan University, Changsha, China; ^2^ GMU-GIBH Joint School of Life Sciences, The Guangdong-Hong Kong-Macau Joint Laboratory for Cell Fate Regulation and Diseases, Guangzhou National Laboratory, Guangzhou Medical University, Guangzhou, China; ^3^ Guangzhou National Laboratory, Guangzhou Medical University, Guangzhou, China

**Keywords:** virus infection, bioinformatics, virome, systemic lupus erythematosus, virus-disease interaction

## Abstract

**Introduction:**

This study aimed to investigate the virome diversity of the SLE disease and the association between viral infections and the disease.

**Methods:**

SLE-related RNA-Seq data were retrieved from public databases. A rigorous computational workflow was employed to identify the human viruses. Differential expression analysis and functional enrichment analysis were conducted in R.

**Results:**

We identified ten human virus species from 826 RNA-Seq samples of human blood, comprising 688 SLE patients and 138 healthy controls. Eight of the ten virus species exhibited higher positive rates in SLE patients compared to healthy controls, with Human betaherpesvirus 5 (HHV5) having the highest positive rate (4.1%) and being exclusively detected in SLE samples. The virus abundances were low and comparable in both SLE patients and healthy controls. Analysis of the antiviral interferon-stimulated genes (ISGs) in samples showed higher ISG expression levels in HHV4 and HHV5-positive samples compared to virus-negative samples. Several genes that were up-regulated in SLE patients were further up-regulated after HHV5 infection, and they were mainly enriched in immune response-related biological processes. Additionally, the expression levels of several marker genes of SLE severity were compared between HHV5-positive and virus-negative SLE patients, suggesting that HHV5 infection may be associated with aggravated SLE disease.

**Discussion:**

We found that SLE patients are more susceptible to viral infections than healthy individuals. Viral infections, such as HHV5, may be associated with aggravated SLE disease. This study deepens our understanding of the association between viruses and SLE and provides new insights into prevention and control of the disease.

## Introduction

Systemic lupus erythematosus (SLE) is a multi-system autoimmune disease with most patients being women of childbearing age. The incidence rate in women is approximately ten times higher than that in men (Lazzaroni et al., 2016). SLE is characterized by the simultaneous dysregulation of innate and adaptive immune systems, leading to the production of various pathogenic autoantibodies and activation of the type I interferon pathway (Fortuna and Brennan, 2013; Nunez et al., 2023). It is a heterogeneous connective tissue disease which can involve almost all organ systems (Bourg et al., 2024). Frequently observed complications in SLE patients include hematological manifestations such as lymphopenia, leukopenia, thrombocytopenia, and hemolytic anemia, as well as anti-phospholipid syndrome and neuropsychiatric manifestations (Aringer et al., 2024). Some of these symptoms may result from either bone marrow failure or excessive peripheral cell destruction, both of which are immune-mediated (Velo-García et al., 2016). The immunopathogenesis of SLE is highly complex, involving three primary mechanisms: self-antigen excess, defects in apoptosis, and inappropriate activation of type I interferon responses. Additionally, drugs and infections are recognized as significant potential triggers for SLE. Despite reports identifying several genetic regions associated with SLE, the exact pathogenesis of the disease remains unclear (Morand et al., 2023).

Viruses have been reported to play an important role in several autoimmune diseases, such as SLE, type 1 diabetes, rheumatoid arthritis, Sjögren’s syndrome, herpetic stromal keratitis, celiac disease, and multiple sclerosis (Jung and Suh, 2017; Posso-Osorio et al., 2021; Zebardast et al., 2023). Infections of multiple viruses including Coxsackie B virus (CVB), rotavirus, influenza A virus (IAV), and herpesvirus, have been proposed to modulate the induction and development of autoimmune diseases (Janahi et al., 2018; Smatti et al., 2019). Many studies have investigated the relationship between SLE and viruses. For example, Stearrett et al. identified differentially expressed human endogenous retroviruses in SLE patients (Stearrett et al., 2021); Pan et al. reported strong associations between SLE and several viruses including Epstein-Barr virus (EBV or HHV4), parvovirus B19 (B19V) and cytomegalovirus (CMV or HHV5) (Pan et al., 2019); Guo et al. identified a large number of viruses in the peripheral blood mononuclear cells (PBMCs) of 10 SLE patients (Guo et al., 2020). Despite these studies, the virome diversity in the SLE disease and the mechanisms underlying the interactions between viral infection and SLE are still unclear.

With the development of next-generation-sequencing technology, virome studies have identified many viruses in humans. For example, Kumata et al. identified 39 animal viruses in 51 somatic tissues of healthy people by analyzing 8991 samples from the GTEx project (Kumata et al., 2020). Ye et al. built the Human Virus Database which included more than 1000 animal viruses observed in 68 human tissues (Ye et al., 2022). Although virome diversity has been extensively explored, viral contributions to human health and disease are still often overlooked. For example, lots of evidence indicated that persistent human cytomegalovirus infection is associated with atherosclerosis and coronary artery disease (Ji et al., 2012); the severe acute respiratory syndrome coronavirus 2 (SARS-CoV-2) has been reported to potentially cause multiple diseases such as placenta diseases, endocarditis, memory disorders and so on (Drago et al., 2021; Desai et al., 2022; Zayet et al., 2022; Piccioni et al., 2023). Thus, it is crucial to identify viruses associated with human diseases to better understand the pathogenic mechanisms underlying these diseases. For example, Kim et al. identified two viruses (picobirnavirus and tobamovirus) which were more prevalent in pregnant women with T1D than in healthy ones (Wook Kim et al., 2019); Deng et al. identified 9 viruses in brain tissues of the Parkinson’s disease (PD) patients and found higher positive rate of most viruses in PD patients compared to healthy individuals (Deng et al., 2023). Unfortunately, most published virome studies on human diseases do not include further investigations into the interactions between the virome and the diseases.

This study aimed to investigate the virome diversity of the SLE disease and the association between viral infections and the disease. First, we collected a large number of SLE-related blood transcriptome datasets from public databases and identified the virome from the data; second, the virome was validated by the analysis of the expression level of antiviral interferon-stimulated genes (ISGs) in samples; third, we identified human genes related to the interaction between SLE and viral infections and analyzed their functions; finally, we analyzed the expression levels of several marker genes related to the severity of SLE in SLE samples. Overall, this study systematically investigated the virome in the SLE disease and the complex interaction between viral infection and the disease, which provides new insights into the pathogenesis mechanisms of SLE.

## Materials and methods

### Data collection

The SLE-related RNA-Seq data were retrieved from the NCBI Sequence Read Archive (SRA) database by the steps described as follows. Firstly, we searched the SRA database using “SLE” or “systemic lupus erythematosus” as terms and kept all transcriptome sequencing samples related to SLE, which resulted in 44 projects and 2,020 runs. Subsequently, we conducted a manual inspection of all samples, retaining only those derived from blood tissue, resulting in 826 samples from 15 projects, which included 688 and 138 samples from SLE patients and healthy controls, respectively (**Supplementary Table S1**).

### Virus identification

A computational workflow was developed to identify viruses from the RNA-Seq data. Firstly, fastp (version=0.20.0) was used to trim adapters and filter low-quality reads. The remaining reads were aligned to the human genome hg38 with bwa (version=0.7.12-r1039). We then queried the unaligned reads using BLASTN (version=2.5.0) against a library of genomic sequences from animal viruses with hosts registered as invertebrates, vertebrates, or humans. The reads with an E-value of less than 1E-10 for the best hit were labeled as hypothetical viral reads. Finally, the putative viral reads were queried against the NT database (downloaded on October 12^th^, 2022) with BLASTN to remove false positives. We retained reads with the best match (largest bit-score) to animal viruses as true viral reads. In cases where there are multiple best matches with the same bit-score, the reads were removed if any of these best matches belongs to non-viral genomes.

### Removal of low-confidence viral reads

The low-reliability viral reads were further removed in the following three aspects: Firstly, the viruses with fewer than three reads mapped and detected only in one dataset were removed; secondly, we removed viruses that have not been reported to infect humans; thirdly, viruses of the Retroviridae family were removed because there is a possibility of cross-contamination from endogenous retroviral sequences, and viruses of the Baculoviridae family were also removed as they are commonly used in the laboratory (Ye et al., 2022).

### Calculation of virus sequence depth

Since the RNA-Seq data used in this study were not originally designed for virus discovery, which could result in an underestimation of virus abundance in humans, the viral abundance in a sample was normalized as reads per kilobase per billion mapped reads (RPKB) according to our previous study (Deng et al., 2023). Specifically, it was calculated as follows:


Viralabundance(RPKB)=M∗10^9/(N∗L)

where M is the number of RNA-Seq reads assigned to a certain virus, N is the total number of RNA-Seq reads assigned to the human genome, and L is the length of the viral genome in base pairs.

### Differential expression analysis of human genes

Raw sequencing reads were quality-controlled as previously described. The resulting clean data were mapped to the human genome hg38 using bwa (version=0.7.12-r1039) with default parameters, and BAM files were sorted with Samtools (version=1.9). Gene counts were obtained using the featureCounts program (version= 2.0.1). The batch-corrected gene expression matrix was generated using the “removeBatchEffect” function in the limma package (version=3.46.0) in R, producing log2 normalized count. Differentially expressed genes (DEGs) between two groups were identified using the “DESeq” function of the DESeq2 package (version=1.30.1) in R. Genes with at least two-fold changes of expressions and the adjusted p-value < 0.05 were considered as DEGs.

### Functional enrichment analysis

Functional enrichment of human genes was performed using the Gene Ontology (GO) and Kyoto Encyclopedia of Genes and Genomes (KEGG) pathway analysis by the clusterProfiler package (version=3.18.1) in R. All KEGG pathways and GO terms with FDR adjusted p-values less than 0.05 were considered significant enrichment.

### Gene set enrichment analysis

GSEA was performed using the R packages fgsea (version=1.16.0) and clusterProfiler (version=3.18.1). The ranked gene list was generated from log fold change (logFC) values of differentially expressed genes. Genes were sorted in descending order based on their logFC values. Custom gene sets were defined using non-SLE-related antiviral ISGs. A permutation test was conducted with 10,000 permutations to assess statistical significance.

### Statistical analysis

All statistical analyses were conducted in Python (version 3.7.9). The Wilcoxon Rank Sum Test was conducted using the “scipy.stats.ranksums” function. Logistic regression analysis was used to evaluate the impact of age and sex on virus positivity and SLE status using the statsmodels.api library. Age and sex were identified as potential confounders and included in the regression model to adjust for their effects. A p-value of smaller than 0.05 was considered statistical significant in all analysis of the study.

## Results

### Overall workflow of the study

**Figure 1:**
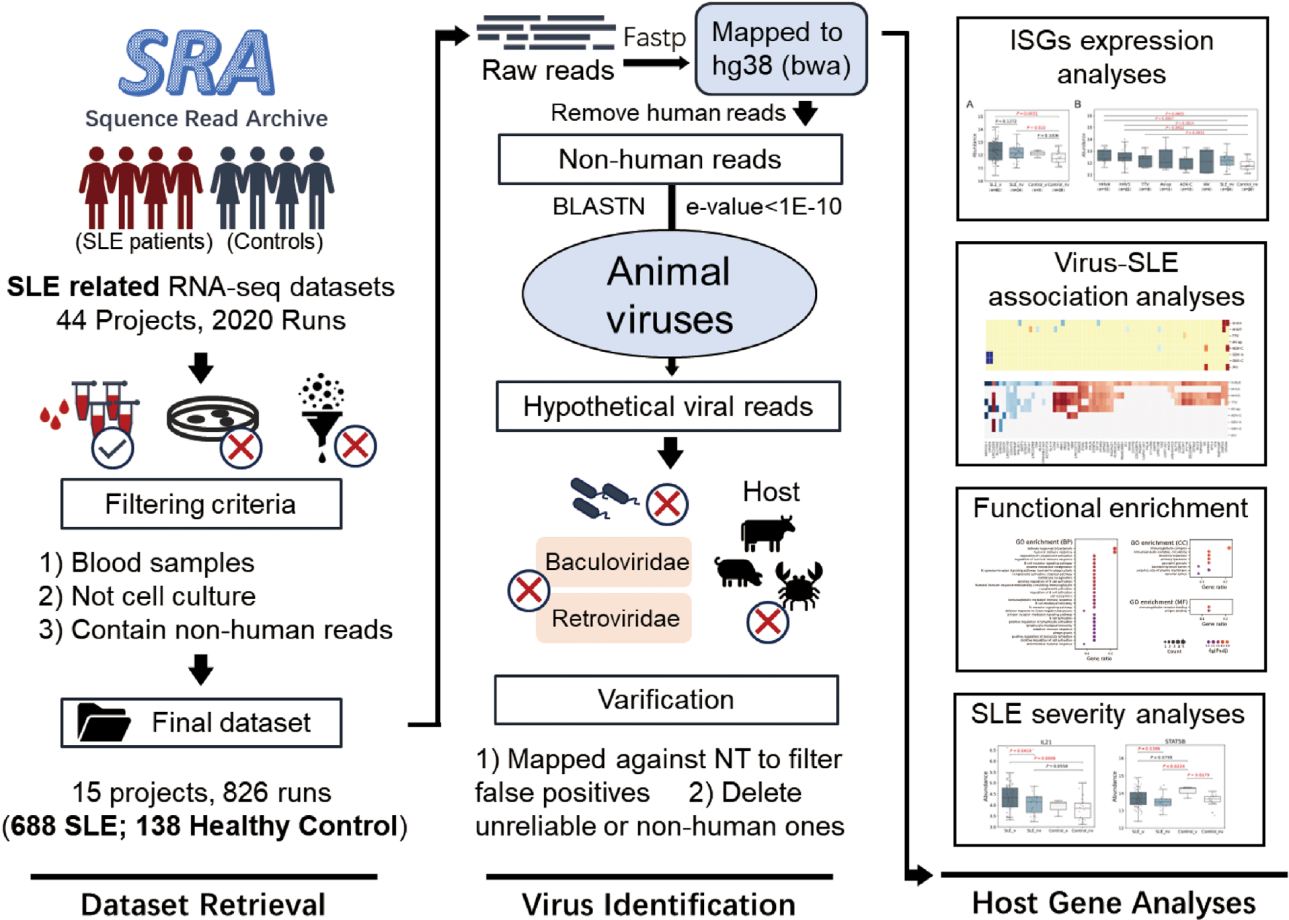
Graphical workflow of the study. It contained three sections: Dataset Retrieval, Virus Identification and Host Gene Analysis.

The workflow of the study is shown in **Figure 1** and contains three sections. The first section was “Dataset Retrieval”, during which 826 samples of human blood including 688 from SLE patients and 138 from healthy controls with RNA-Seq data were obtained from 15 datasets in the NCBI SRA database. The second section was “Virus Identification”, during which human viruses were identified from these samples using a homology-based method, and their abundances were quantified based on the RNA-Seq data. The third section, titled “Host Gene Analysis”, involved quantifying human gene expression profiles and using them to analyze ISG expressions, the interactions between SLE and viral infections, and SLE severity.

### Ten human viruses were identified with low abundances and low positive rates

**Figure 2:**
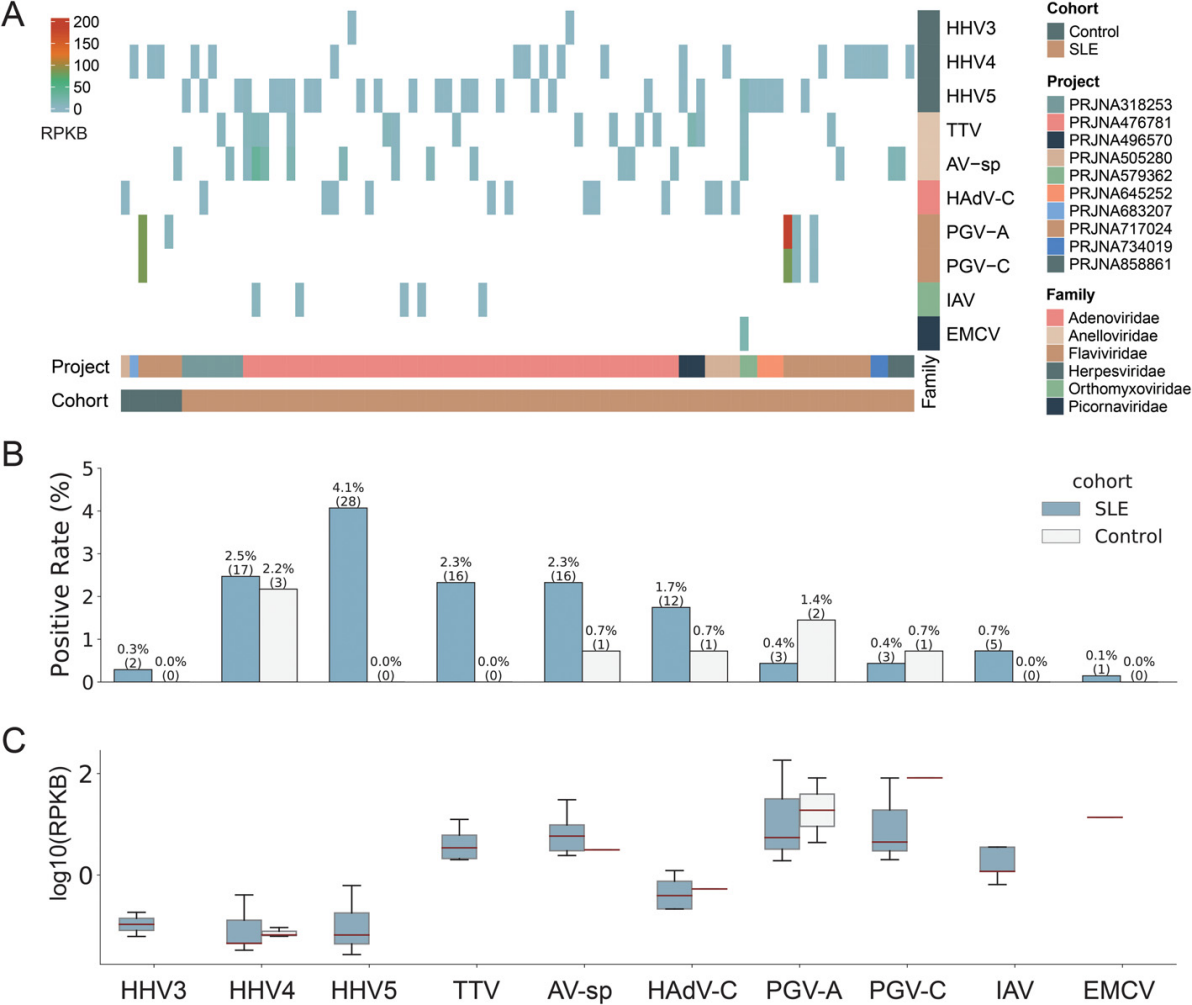
Overview of the virome identified in the study. **(A)** The abundance of viruses identified in positive samples. Each column represents a sample with the project and cohort information tagged by the color bars at the bottom. **(B)** The comparison of virus positive rates between SLE and Control groups. The numbers of positive samples were tagged in the brackets. **(C)** The comparison of virus abundance between SLE and Control groups. For clarity, virus names were shown as abbreviations. The full names and taxonomy IDs of these viruses were shown in **Supplementary Table S4**.

Among all 826 human blood samples mentioned above, a total of 10 human viruses, including three viruses from the Herpesviridae family (Human alphaherpesvirus 3 (HHV3), Human gammaherpesvirus 4 (HHV4), Human betaherpesvirus 5 (HHV5)), two viruses from the Anelloviridae family (Torque teno virus (TTV), Anelloviridae sp. (AV-sp)), two viruses from the Flaviviridae family (Human pegivirus (PGV-A), GB virus C (PGV-C)), one virus from the Adenoviridae family (Human mastadenovirus C (HAdV-C)), one virus from the Orthomyxoviridae family (Influenza A virus (IAV)) and one virus from the Picornaviridae family (Cardiovirus A (EMCV)), were identified in 84 SLE patients and 7 healthy controls (**Figure 2A** & **Supplementary Table S2**). Most virus-positive samples (n=76) contained only one virus; in the remaining ones (n=8), 2~4 viruses were identified. Half of the viruses were identified in fewer than 10 samples. HHV5 was the most commonly detected virus and was identified in 28 samples, accounting for 3.4% of all samples, while EMCV was only detected in one sample (**Figure 2B**). Analysis of the virus abundance in blood samples showed that the majority of viruses had low abundances, with the exception of pegivirus (PGV-A and PGV-C in **Figure 2C**), which has been reported to be widely distributed in human populations and has long-term infections in humans (Cebriá-Mendoza et al., 2021).

We then assessed the impact of potential confounders including age and sex on virus positivity (**Supplementary Table S3**). The results showed that both age (p-value = 0.335) and sex (p-value=0.067) did not significantly affect virus positivity. These findings suggest limited influence of age and sex on the virus identification in the study.

### SLE patients had higher positive rates of viruses than healthy controls

We then compared the viromes identified from SLE patients and healthy controls. All ten viruses mentioned above were identified in SLE patients, while only five of them (HHV4, AV-sp, HAdV-C, PGV-A, and PGV-C) were identified in healthy controls. Regarding positive rates, all viruses, except for PGV-A and PGV-C, had higher rates in SLE patients than in healthy controls, although the positive rates of all viruses were less than 5% (**Figure 2B**). For example, both HHV5 and TTV were only detected in SLE patients with a positive rate of 4.1% and 2.3%, respectively. When taking all viruses together, SLE patients exhibited a virus-positive rate more than twice that in healthy controls (12.2% vs 5.1%). Most viruses had similar or lower abundances in SLE patients than in healthy controls. Interestingly, both PGV-A and PGV-C had higher positive rates and abundances in healthy controls than in SLE patients (**Figures 2B, C**). To sum up, viruses identified from SLE patients showed greater diversity and higher positive rates than those from healthy controls.

### Higher ISG expression levels in HHV4 and HHV5-positive samples

**Figure 3:**
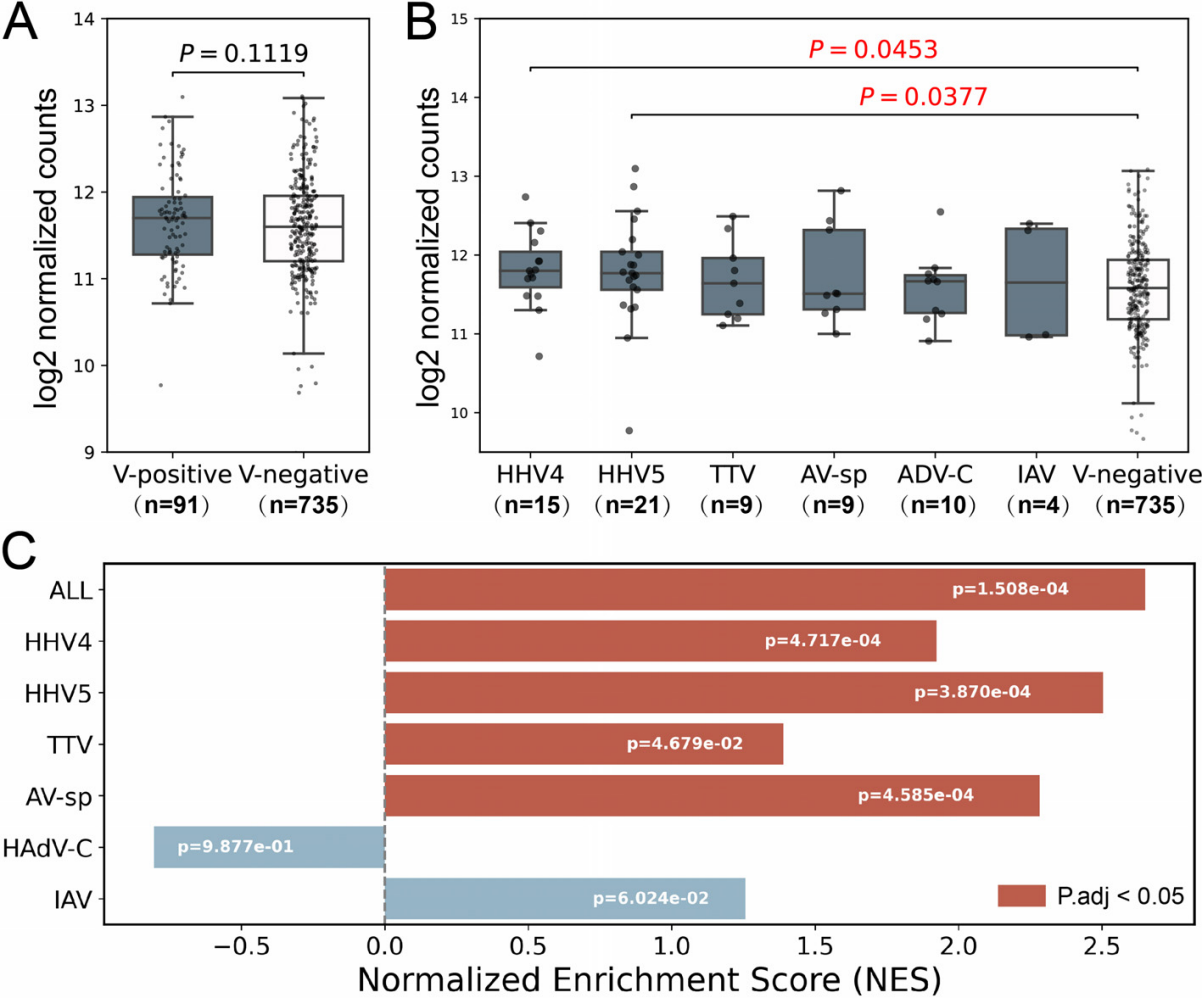
Analysis of expression level of non-SLE-related antiviral ISGs in samples used in the study. **(A)** The comparison of expression levels of non-SLE-related antiviral ISGs between virus-positive samples and virus-negative samples. **(B)** The comparison of expression levels of non-SLE-related antiviral ISGs in virus-positive samples grouped by virus to that in virus-negative samples. P-values based on Wilcoxon rank sum test were tagged. **(C)** GSEA analyses of non-SLE-related antiviral ISGs. Positive and negative NES values indicate higher and lower expression, respectively, in virus-positive SLE samples compared to virus-negative SLE samples. Samples which were found to contain two or more viruses were excluded in the analysis. Only six viruses were analyzed as less than 3 samples were positive for the other four viruses. The numbers of samples in each group were shown in brackets.

The Type I interferon (IFN-I) response is crucial in combating viral infections, primarily by inducing the expression of ISGs that possess antiviral functions (Cao et al., 2023). It also plays an important role in the development of SLE disease, and the expression of certain ISGs is increased in SLE patients (Monaghan et al., 2023). To validate the virome identified in the study, a set of antiviral ISGs were obtained from Zhou’s study (Zhou et al., 2020), and a set of SLE-related ISGs were obtained from Siddiqi’s study (**Supplementary Table S5**) (Siddiqi et al., 2021). As expected, the expression level of SLE-related ISGs were higher in SLE patients than in non-SLE patients (**Supplementary Figure S1**). Then, the SLE-related ISGs were excluded from the antiviral ISGs to remove the influence of SLE on ISG expressions, leading to a set of non-SLE-related antiviral ISGs. The expression levels of non-SLE-related antiviral ISGs were analyzed to confirm the presence of viral infection in virus-positive samples. As shown in **Figure 3A**, the non-SLE-related antiviral ISGs had slightly higher expressions in virus-positive samples than in virus-negative ones. Further analysis of these genes in virus-positive samples grouped by virus (**Figure 3B**) showed that for all viruses except AV-sp, the median expression levels of non-SLE-related antiviral ISGs in virus-positive groups were higher than those in virus-negative Control group, although statistical differences were only observed for HHV4 (p-value=0.0453) and HHV5 (p-value=0.0377). In addition, we conducted GSEA analysis on the non-SLE-related antiviral ISGs in virus-positive samples to further illustrate the upregulation of these genes in virus-infected samples. As shown in **Figure 3C**, significant up-regulation of ISGs was observed in virus-positive samples as a total and in four of six viruses analyzed.

### The immune system was over-activated in SLE patients with HHV5 infection

**Figure 4:**
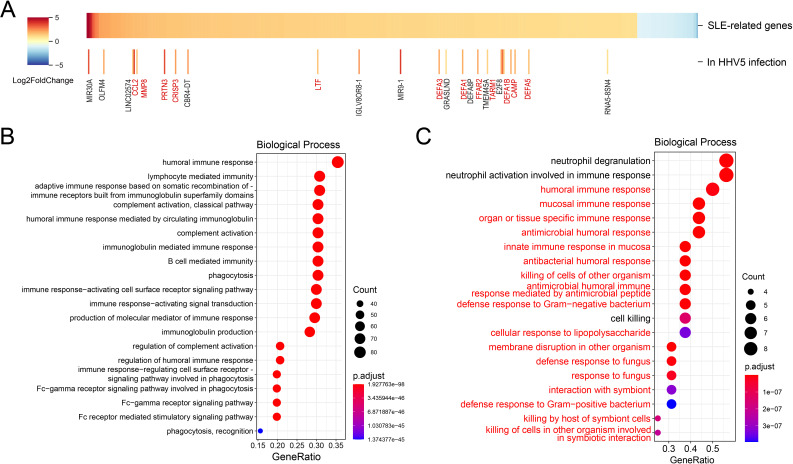
Analyses of the interactions between the SLE disease and HHV5 infection. **(A)** Differential analysis of SLE-related genes between HHV5-positive and virus-negative SLE samples. The upper panel shows differentially expressed genes based on fold changes. The lower panel highlights SLE-related genes that exhibit further significant expression changes in HHV5 infection. Genes related to the immune response are highlighted in red to emphasize their relevance to disease pathology. **(B)** Top 20 enriched biological processes for up-regulated SLE-related genes. **(C)** Top 20 enriched biological processes for further up-regulated SLE-related genes in HHV5-positive SLE samples. The biological processes that were related to immune responses and were also enriched in all SLE-related genes were highlighted in red.

Then, we investigated the interaction between the SLE disease and HHV5 infection since HHV5 showed significantly higher positive rate and ISG expression levels than healthy controls. Firstly, the differentially expressed human genes in virus-negative SLE samples compared to virus-negative control samples were identified to investigate the influence of the SLE on human gene expressions. A total of 463 up-regulated and 52 down-regulated DEGs were identified and defined as SLE-related genes (**Figure 4A**). The up-regulated SLE-related genes were mainly enriched in terms related to humoral immunity, immunoglobulin and phagocytosis, with “humoral immune response” being the most enriched biological process (**Figure 4B**). Meanwhile, no significantly enriched functions were obtained for the down-regulated SLE-related genes.

To investigate the influence of HHV5 infection on the SLE disease, the DEGs in HHV5-positive SLE samples compared to virus-negative SLE samples were identified. As shown in **Figure 4A**, expression levels of most SLE-related genes were not significantly changed by HHV5 infection. Interestingly, for the 23 genes with significant changes, all of them were up-regulated in HHV5 infection, and more than half of them (n=12) were related to immune response (highlighted in red in **Figure 4A**). For example, CAMP plays an important role in innate immunity defense against viruses, and LTF is an important component of the non-specific immune system. Analysis of functions of the further-up-regulated genes showed that they were mainly enriched in biological processes related to immune responses, such as “neutrophil degranulation”, “neutrophil activation involved in immune response”, “humoral immune response”, and so on. 17 of the top 20 enriched biological processes were also observed for the SLE-related genes (highlighted in red in **Figure 4C**).

### HHV5 infection was associated with aggravated SLE disease

**Figure 5:**
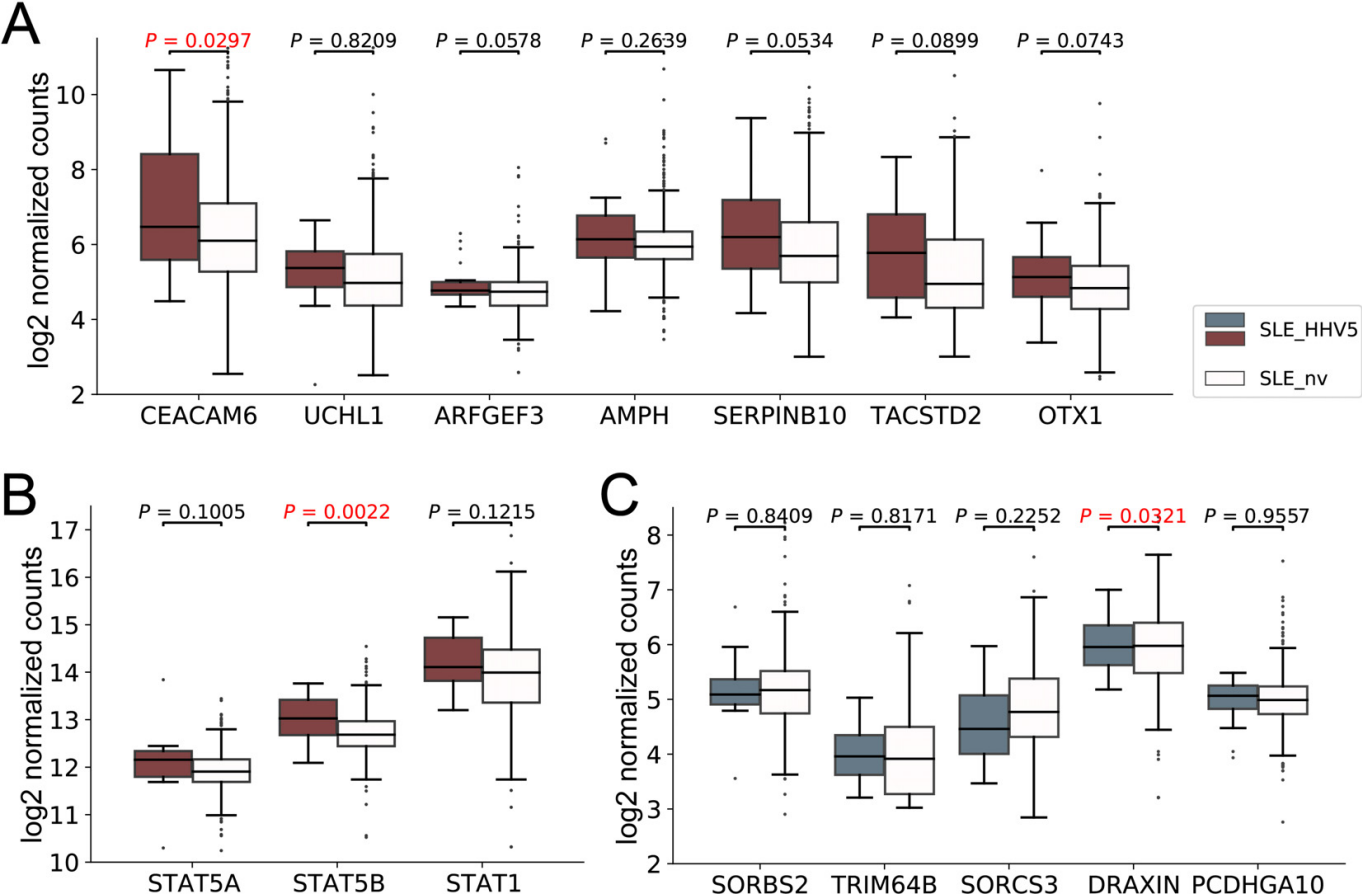
The expression level of SLE severity marker genes in HHV5-positive SLE patients (SLE_HHV5, colored boxes) and virus-negative SLE patients (SLE_nv, plain boxes). **(A)** The expression level of seven severe SLE up-regulated DEGs collected by Zhang et al. **(B)** The expression level of three severe SLE marker genes collected by Goropevšek et al. **(C)** The expression level of five severe SLE down-regulated DEGs collected by Zhang et al. Red box plots represent genes known to have higher expression levels in severe SLE, while blue box plots represent those with lower expression levels in severe SLE. *P-*values based on Wilcoxon rank sum test were tagged.

Given that the immune system was over-activated in HHV5-positive SLE patients, it is suspected that the HHV5 infection may be associated with aggravated SLE disease. Therefore, we firstly collected genes known to be associated with the severity of SLE. This included 7 upregulated (CEACAM6, UCHL1, ARFGEF3, AMPH, SERPINB10, TACSTD2, and OTX1) and 5 downregulated (SORBS2, TRIM64B, SORCS3, DRAXIN, and PCDHGA10) DEGs in the severe SLE group compared to mild SLE group identified in Zhang’s study (Zhang et al., 2023), and two genes (STAT1 and STAT5) that showed elevated expressions in the aggravation of the SLE disease in studies of Goropevšek et al. and Aue et al (Goropevšek et al., 2019; Aue et al., 2020). Then, we compared the expression levels of these genes in our HHV5-positive and virus-negative SLE samples (**Figure 5**). As expected, all nine upregulated genes associated with severe SLE had higher median expressions in HHV5-positive SLE group than in HHV5-negative SLE group, although statistical significance was only observed for two genes (CEACAM6, p-value = 0.0297; STAT5B, p-value = 0.0022) (**Figures 5A, B**). For five downregulated genes associated with severe SLE, three genes had lower median expressions in HHV5-positive SLE group than in HHV5-negative group, although statistical significance was only observed for the gene DRAXIN (p-value = 0.0321) (**Figure 5C**).

## Discussion

In this study, we collected the most abundant blood transcriptome sequencing data of the SLE disease so far, and conducted rigorous identification of human viruses, obtaining a comprehensive virus profile in the blood of SLE patients. A total of ten human virus species were detected with low positive rates and low abundances in SLE patients; however, the positive rates of viruses were higher in the SLE patients than those in healthy controls, suggesting that the SLE patients may be more susceptible to viral infections than healthy people, which was consistent with previous studies (Quaglia et al., 2021). Analysis of antiviral ISG expression level confirms viral infections in virus-positive samples, further validating the virome identified in our study.

Previous studies by Guo et al. identified 36 human-associated viruses (not at the species level) from the RNA-Seq data of the PBMCs of 10 SLE patients (Guo et al., 2020). In contrast, our study only identified ten human virus species from more than 600 samples of SLE patients. The difference was mainly due to the different workflows used for virus identification. Guo et al. identified viruses by firstly removing all reads aligned to the human genome and then by querying against RefSeq virus genomes with the unmapped reads using BLASTN. All reads that had an E-value smaller than 1E-5 in the BLAST were taken as viral reads. Compared to their workflow, we used a more reliable strategy (Materials and Methods): i) only reads which had an E-value of smaller than 1E-10 in the BLAST were taken as hypothetical viral reads; ii) the hypothetical viral reads were further queried against the NCBI NT database to remove false positives; iii) low-reliability viruses were further removed due to low abundance or potential contamination. Besides, viral infections of samples were confirmed by analysis of the ISG expressions. Thus, although far fewer viruses were identified, the virome identified in our study was much more reliable than that in Guo’s study.

The ten virus species identified in our study have been commonly reported in humans: i) Three viruses from the Herpesviridae family (HHV3, HHV4, and HHV5) have frequently been reported in humans with a high positive rate (Janahi et al., 2018; Ewbank et al., 2023; Fang et al., 2023). Among them, HHV4 is considered to be definitely associated with SLE (Pan et al., 2019), and researchers have observed elevated SLEDAI (Systemic Lupus Erythematosus Disease Activity Index) scores in patients with lytic HHV4 infections (Han et al., 2018). HHV5 is also believed to contribute significantly to the development of SLE, as multiple studies have indicated a higher prevalence of HHV5 among SLE patients (Mohamed et al., 2015; Pan et al., 2019). Notably, our identification results revealed that HHV4 and HHV5 exhibited the highest positive rate in SLE patients, with HHV5 exclusively detected in SLE samples. This further substantiates their strong association with SLE. Subsequent analyses focusing on ISG expression and markers of SLE severity highlighted the most prominent differences between HHV5-positive samples and virus-negative samples, thus reinforcing the substantial impact of HHV5 infection on SLE. ii) TTV and AV-sp, which exhibited relatively low positive rates, are categorized as non-pathogenic anelloviruses that are believed to be widely present in the human blood virome (Cebriá-Mendoza et al., 2021). It is noteworthy that multiple studies have reported a higher positive rate of anelloviruses in SLE patients compared to that in healthy people (Costa et al., 2012; Quaglia et al., 2021). iii) Two members of the Flaviviridae family, PGV-A and PGV-C, are also generally regarded as non-pathogenic. Compared to anelloviruses, most researchers believe that their population distribution is relatively smaller (Cebriá-Mendoza et al., 2021), which is consistent with our results. iv) HAdV-C was indicated by a higher prevalence in SLE patients in our results, despite its low detection rate in the general population (Guo et al., 2022). v) As for IAV and EMCV, limited reports suggest their potential connection with autoimmune diseases, although the IAV is one of the most prevalent respiratory viruses in human populations (He et al., 2014).

SLE patients have concurrent dysregulation of innate and adaptive immune systems (Fortuna and Brennan, 2013). Our study revealed the increased expressions of SLE-related ISGs in SLE patients. Most SLE-related genes were up-regulated and primarily enriched in immune-related biological processes. Interestingly, certain up-regulated genes in SLE patients were further up-regulated in HHV5 infection, primarily involved in immune responses. This finding aligns with a previous study by Han et al., which reported the evaluated expression of specific ISGs in SLE patients following HHV4 infections (Han et al., 2018). This suggests that infections of several viruses such as HHV5 and HHV4 may trigger an over-activated immune response, potentially exacerbating the SLE disease. This was partially validated by our findings, which showed that all severity marker genes of SLE which were up-regulated in severe SLE patients had higher median expressions in HHV5-positive SLE samples than in virus-negative SLE samples. These findings suggest the importance of timely monitoring the viral infection status of SLE patients and preventing viral infections during the treatment of the disease. This proactive approach may effectively reduce the likelihood of rapid disease progression or deterioration.

There were some limitations to the study. Firstly, it is difficult to establish the causal relationship between HHV5 infection and aggravated SLE disease based on current analysis. Longitudinal studies and animal models are needed to build such a relationship (Pisetsky, 2023). Secondly, only 688 SLE samples were used in the analysis, and the RNA-Seq data of these samples in the original studies were not designed for virus discovery, which may lead to the underestimation of the virus diversity and abundance in SLE diseases. Many more studies such as large-scale RNA-Seq or single-cell sequencing of multiple tissues in SLE patients are needed to uncover the virome diversity in SLE disease. Thirdly, when analyzing differential gene expression, it is difficult to separate the effect of SLE disease and the infection of specific viruses, as there were few virus-positive healthy controls for each virus. Further investigations with more blood samples may improve the study.

## Conclusions

This is the first study to systematically investigate the virome in the SLE disease and the complex interaction between viral infections and the disease. Ten viruses were identified in the blood of SLE patients with low abundances. Besides, the HHV5 infection may be associated with aggravated SLE disease. Our findings contribute to the understanding of the association mechanism between SLE disease and viral infections, and also provide new clues for the prevention and control of the disease.

## Data Availability

The original contributions presented in the study are included in the article/**Supplementary Material**. Further inquiries can be directed to the corresponding authors.

## References

[B1] Aringer M. Toro-Domínguez D. Alarcón-Riquelme M. E. (2024). Classification of systemic lupus erythematosus: From the development of classification criteria to a new taxonomy? Best Pract. Res. Clin. Rheumatol. 37 (4), 101949. doi: 10.1016/j.berh.2024.101949 38729901

[B2] Aue A. Szelinski F. Weißenberg S. Wiedemann A. Rose T. Lino A.. (2020). Elevated STAT1 expression but not phosphorylation in lupus B cells correlates with disease activity and increased plasmablast susceptibility. Rheumatol. (Oxford England) 59, 3435–3442. doi: 10.1093/rheumatology/keaa187 32357246

[B3] Bourg C. Le Tallec E. Curtis E. Lee C. Bouzillé G. Oger E.. (2024). Heterogeneity of right ventricular echocardiographic parameters in systemic lupus erythematosus among four clinical subgroups, as stratified by clinical organ involvement in observational cohort. Open Heart 11, e002615. doi: 10.1136/openhrt-2024-002615 38702088 PMC11086574

[B4] Cao Z. Liu C. Peng C. Ran Y. Yao Y. Chiu S.. (2023). Ebola virus VP35 perturbs type I interferon signaling to facilitate viral replication. Virologica Sin. 38(6), 922–930. doi: 10.1016/j.virs.2023.10.004 PMC10786653 37839549

[B5] Cebriá-Mendoza M. Bracho M. A. Arbona C. Larrea L. Díaz W. Cuevas J. M.. (2021). Exploring the diversity of the human blood virome. Viruses 13, 2322. doi: 10.3390/v13112322 34835128 PMC8621239

[B6] Costa M. R. Costa I. P. Devalle S. Castro A. R. Freitas S. Z. (2012). Prevalence and genetic diversity of torque teno virus in patients with systemic lupus erythematosus in a reference service in Mato Grosso do Sul. Rev. Bras. reumatologia 52, 49–54. 22286645

[B7] Deng L. Fu P. Ding L. Duan X. Feng S. Peng Y. (2023). Virome analysis provides new insights into the association between viruses and Parkinson’s disease. J. Med. Virol. 95, e28111. doi: 10.1002/jmv.28111 36042689

[B8] Desai A. D. Lavelle M. Boursiquot B. C. Wan E. Y. (2022). Long-term complications of COVID-19. Am. J. Physiol. Cell Physiol. 322, C1–c11. doi: 10.1152/ajpcell.00375.2021 34817268 PMC8721906

[B9] Drago F. Ciccarese G. Merlo G. Trave I. Javor S. Parodi A.. (2021). Oral and cutaneous manifestations of viral and bacterial infections: Not only COVID-19 disease. Clinics Dermatol. 39, 384–404. doi: 10.1016/j.clindermatol.2021.01.021 PMC7849469 34517997

[B10] Ewbank A. C. Duarte-Benvenuto A. Zamana-Ramblas R. Sacristán I. Costa-Silva S. Sacristán C.. (2023). Herpesvirus and adenovirus surveillance in threatened wild West Indian (Trichechus manatus) and Amazonian manatees (Trichechus inunguis), Brazil. Acta tropica 237, 106740. doi: 10.1016/j.actatropica.2022.106740 36332674

[B11] Fang L.-Z. Dong Y. H. Yan Z. J. Zhou C. M. Yu X. J. Qin X. R. (2023). Reactivation of Epstein-Barr virus in SFTSV infected patients. Infect. Med. 2, 195–201. doi: 10.1016/j.imj.2023.04.005 PMC10699715 38073887

[B12] Fortuna G. Brennan M. T. (2013). Systemic lupus erythematosus: epidemiology, pathophysiology, manifestations, and management. Dental Clinics North America 57, 631–655. doi: 10.1016/j.cden.2013.06.003 24034070

[B13] Goropevšek A. Holcar M. Pahor A. Avčin T. (2019). STAT signaling as a marker of SLE disease severity and implications for clinical therapy. Autoimmun. Rev. 18, 144–154. doi: 10.1016/j.autrev.2018.08.010 30572141

[B14] Guo G. Ye L. Shi X. Yan K. Huang J. Zhang H.. (2020). Dysbiosis in peripheral blood mononuclear cell virome associated with systemic lupus erythematosus. Front. Cell. infection Microbiol. 10. doi: 10.3389/fcimb.2020.00131 PMC7153479 32328467

[B15] Guo J. Huang X. Zhang C. Huang P. Li Y. Cao Y.. (2022). The blood virome of 10,585 individuals from the ChinaMAP. Cell Discovery 8, 113. doi: 10.1038/s41421-022-00476-1 36253359 PMC9576698

[B16] Han L. Zhang Y. Wang Q. Xin M. Yang K. Lei K.. (2018). Epstein-Barr virus infection and type I interferon signature in patients with systemic lupus erythematosus. Lupus 27(6), 947–954. doi: 10.1177/0961203317753069 29338588

[B17] He Y. Lin G. Y. Wang Q. Cai X. Y. Zhang Y. H. Lu X. D.. (2014). A 3-year prospective study of the epidemiology of acute respiratory viral infections in hospitalized children in Shenzhen, China. Influenza other Respir. viruses 8, 443–451. doi: 10.1111/irv.12257 24828783 PMC4181804

[B18] Janahi E. M. A. Das S. Bhattacharya S. N. Haque S. Akhter N. Dar S. A.. (2018). Cytomegalovirus aggravates the autoimmune phenomenon in systemic autoimmune diseases. Microbial pathogenesis 120, 132–139. doi: 10.1016/j.micpath.2018.04.041 29704668

[B19] Ji Y. N. An L. Zhan P. Chen X. H. (2012). Cytomegalovirus infection and coronary heart disease risk: a meta-analysis. Mol. Biol. Rep. 39, 6537–6546. doi: 10.1007/s11033-012-1482-6 22311014

[B20] Jung J. Y. Suh C. H. (2017). Infection in systemic lupus erythematosus, similarities, and differences with lupus flare. Korean J. Internal Med. 32, 429–438. doi: 10.3904/kjim.2016.234 28490724 PMC5432804

[B21] Kumata R. Ito J. Takahashi K. Suzuki T. Sato K. (2020). A tissue level atlas of the healthy human virome. BMC Biol. 18, 55. doi: 10.1186/s12915-020-00785-5 32493363 PMC7269688

[B22] Lazzaroni M. G. Dall’Ara F. Fredi M. Nalli C. Reggia R. Tincani A.. (2016). A comprehensive review of the clinical approach to pregnancy and systemic lupus erythematosus. J. Autoimmun. 74, 106–117. doi: 10.1016/j.jaut.2016.06.016 27377453

[B23] Mohamed A. E. Hasen A. M. Mohammed G. F. Elmaraghy N. N. (2015). Real-Time PCR of cytomegalovirus and Epstein-Barr virus in adult Egyptian patients with systemic lupus erythematosus. Int. J. rheumatic Dis. 18, 452–458. doi: 10.1111/1756-185x.12261 24341363

[B24] Monaghan K. A. Hoi A. Gamell C. Tai T. Y. Linggi B. Wilson N.. (2023). CSL362 potently and specifically depletes pDCs invitro and ablates SLE-immune complex-induced IFN responses. iScience 26, 107173. doi: 10.1016/j.isci.2023.107173 37456846 PMC10338305

[B25] Morand E. F. Vital E. M. Petri M. van Vollenhoven R. Wallace D. J. Dörner T. (2023). Baricitinib for systemic lupus erythematosus: a double-blind, randomised, placebo-controlled, phase 3 trial (SLE-BRAVE-I). Lancet (London England) 401, 1001–1010. doi: 10.1016/s0140-6736(22)02607-1 36848918

[B26] Nunez D. Patel D. Volkov J. Wong S. Vorndran Z. Basu S.. (2023). Cytokine and reactivity profiles in SLE patients following anti-CD19 CART therapy. Mol. Ther. Methods Clin. Dev. 31, 101104. doi: 10.1016/j.omtm.2023.08.023 37744005 PMC10514439

[B27] Pan Q. Liu Z. Liao S. Ye L. Lu X. Liu H.. (2019). Current mechanistic insights into the role of infection in systemic lupus erythematosus. Biomedicine pharmacotherapy = Biomedecine pharmacotherapie 117, 109122. doi: 10.1016/j.biopha.2019.109122 31226637

[B28] Piccioni A. Franza L. Rosa F. Candelli M. Covino M. La Russa R.. (2023). The role of SARS-COV-2 infection in promoting abnormal immune response and sepsis: A comparison between SARS-COV-2-related sepsis and sepsis from other causes. Infect. Med. 2, 202–211. doi: 10.1016/j.imj.2023.04.006 PMC10699677 38073889

[B29] Pisetsky D. S. (2023). Pathogenesis of autoimmune disease. Nat. Rev. Nephrol. 19, 509–524. doi: 10.1038/s41581-023-00720-1 37165096 PMC10171171

[B30] Posso-Osorio I. Tobón G. J. Cañas C. A. (2021). Human endogenous retroviruses (HERV) and non-HERV viruses incorporated into the human genome and their role in the development of autoimmune diseases. J. Trans. Autoimmun. 4, 100137. doi: 10.1016/j.jtauto.2021.100137 PMC8669383 34917914

[B31] Quaglia M. Merlotti G. De Andrea M. Borgogna C. Cantaluppi V. (2021). Viral infections and systemic lupus erythematosus: new players in an old story. Viruses 13, 277. doi: 10.3390/v13020277 33670195 PMC7916951

[B32] Siddiqi K. Z. Wilhelm T. R. Ulff-Møller C. J. Jacobsen S. (2021). Cluster of highly expressed interferon-stimulated genes associate more with African ancestry than disease activity in patients with systemic lupus erythematosus. A systematic review of cross-sectional studies. Trans. research: J. Lab. Clin. Med. 238, 63–75. doi: 10.1016/j.trsl.2021.07.006 34343626

[B33] Smatti M. K. Cyprian F. S. Nasrallah G. K. Al Thani A. A. Almishal R. O. Yassine H. M.. (2019). Viruses and autoimmunity: A review on the potential interaction and molecular mechanisms. Viruses 11, 762. doi: 10.3390/v11080762 31430946 PMC6723519

[B34] Stearrett N. Dawson T. Rahnavard A. Bachali P. Bendall M. L. Crandall K. A.. (2021). Expression of human endogenous retroviruses in systemic lupus erythematosus: multiomic integration with gene expression. Front. Immunol. 12. doi: 10.3389/fimmu.2021.661437 PMC8112243 33986751

[B35] Velo-García A. Castro S. G. Isenberg D. A. (2016). The diagnosis and management of the haematologic manifestations of lupus. J. Autoimmun. 74, 139–160. doi: 10.1016/j.jaut.2016.07.001 27461045

[B36] Wook Kim K. Allen D. W. Briese T. Couper J. J. Barry S. C. Colman P. G.. (2019). Distinct gut virome profile of pregnant women with type 1 diabetes in the ENDIA study. Open Forum Infect. Dis. 6, ofz025. doi: 10.1093/ofid/ofz025 30815502 PMC6386807

[B37] Ye S. Lu C. Qiu Y. Zheng H. Ge X. Peng Y.. (2022). An atlas of human viruses provides new insights into diversity and tissue tropism of human viruses. Bioinformatics 38, 3087–3093. doi: 10.1093/bioinformatics/btac275 35435220

[B38] Zayet S. Zahra H. Belfeki N. Klopfenstein T. Hagenkötter B. (2022). Atypical miller-fisher syndrome after COVID-19 and sleeve gastrectomy: Contribution of neurochemical markers to early diagnosis. Infect. Med. 1, 140–142. doi: 10.1016/j.imj.2022.02.001 PMC8864887 38073877

[B39] Zebardast A. Hasanzadeh A. Ebrahimian Shiadeh S. A. Tourani M. Yahyapour Y. (2023). COVID-19: A trigger of autoimmune diseases. Cell Biol. Int. 47, 848–858. doi: 10.1002/cbin.11997 36740221

[B40] Zhang X. Zhang J. Pan Z. Zhang Y. Xu X. Wen L.. (2023). Transcriptome sequencing reveals novel molecular features of SLE severity. Front. Genet. 14, 1121359. doi: 10.3389/fgene.2023.1121359 37554401 PMC10406386

[B41] Zhou Z. Ren L. Zhang L. Zhong J. Xiao Y. Wang J.. (2020). Heightened innate immune responses in the respiratory tract of COVID-19 patients. Cell Host Microbe 27, 883–890.e882. doi: 10.1016/j.chom.2020.04.017 32407669 PMC7196896

