# Supplementary material for: Virome analysis provides new insights into the pathogenesis mechanism and treatment of SLE disease
Source: Front Cell Infect Microbiol. 2024 Oct 24;14:1484529. doi: 10.3389/fcimb.2024.1484529 (PMC11540821; doi:10.3389/fcimb.2024.1484529)
Supplement: Supplementary file 1 [file Table1.docx]

**Supplementary Materials for**

**Virome analysis provides new insights into the pathogenesis mechanism and treatment of SLE disease**

**Supplementary Figures**

**Figure S1.** The comparison of SLE-related ISGs between SLE samples and non-SLE samples. *P-*values based on Wilcoxon rank sum test were tagged. The numbers of samples in each group were shown in brackets.


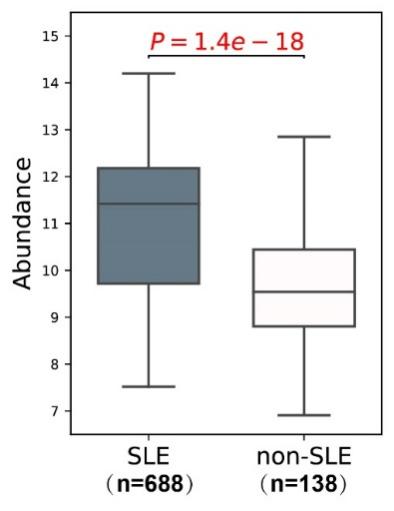


**Supplementary Tables**

**Supplementary Table S1.** The samples used in the study.

|  |  |  |  |  |  |
| --- | --- | --- | --- | --- | --- |
| **Project ID** | **Run** | **Group** | **Body_site** | **Age** | **Sex** |
| PRJNA318253 | SRR3362713 | SLE | Blood | NA | NA |
| PRJNA318253 | SRR3362715 | SLE | Blood | NA | NA |
| PRJNA318253 | SRR3362717 | SLE | Blood | NA | NA |
| PRJNA318253 | SRR3362719 | SLE | Blood | NA | NA |
| PRJNA318253 | SRR3362721 | SLE | Blood | NA | NA |
| PRJNA318253 | SRR3362723 | SLE | Blood | NA | NA |
| PRJNA318253 | SRR3362725 | SLE | Blood | NA | NA |
| PRJNA318253 | SRR3362727 | SLE | Blood | NA | NA |
| PRJNA318253 | SRR3362729 | SLE | Blood | NA | NA |
| PRJNA318253 | SRR3362731 | SLE | Blood | NA | NA |
| PRJNA318253 | SRR3362733 | SLE | Blood | NA | NA |
| PRJNA318253 | SRR3362734 | SLE | Blood | NA | NA |
| PRJNA318253 | SRR3362735 | Control | Blood | NA | NA |
| PRJNA318253 | SRR3362736 | Control | Blood | NA | NA |
| PRJNA318253 | SRR3362737 | Control | Blood | NA | NA |
| PRJNA318253 | SRR3362738 | Control | Blood | NA | NA |
| PRJNA476781 | SRR7367602 | SLE | Blood | NA | NA |
| PRJNA476781 | SRR7367603 | SLE | Blood | NA | NA |
| PRJNA476781 | SRR7367604 | SLE | Blood | NA | NA |
| PRJNA476781 | SRR7367605 | SLE | Blood | NA | NA |
| PRJNA476781 | SRR7367606 | SLE | Blood | NA | NA |
| PRJNA476781 | SRR7367607 | SLE | Blood | NA | NA |
| PRJNA476781 | SRR7367608 | SLE | Blood | NA | NA |
| PRJNA476781 | SRR7367609 | SLE | Blood | NA | NA |
| PRJNA476781 | SRR7367610 | SLE | Blood | NA | NA |
| PRJNA476781 | SRR7367611 | SLE | Blood | NA | NA |
| PRJNA476781 | SRR7367612 | SLE | Blood | NA | NA |
| PRJNA476781 | SRR7367613 | SLE | Blood | NA | NA |
| PRJNA476781 | SRR7367614 | SLE | Blood | NA | NA |
| PRJNA476781 | SRR7367615 | SLE | Blood | NA | NA |
| PRJNA476781 | SRR7367616 | SLE | Blood | NA | NA |
| PRJNA476781 | SRR7367617 | SLE | Blood | NA | NA |
| PRJNA476781 | SRR7367618 | SLE | Blood | NA | NA |
| PRJNA476781 | SRR7367619 | SLE | Blood | NA | NA |
| PRJNA476781 | SRR7367620 | SLE | Blood | NA | NA |
| PRJNA476781 | SRR7367621 | SLE | Blood | NA | NA |
| PRJNA476781 | SRR7367622 | SLE | Blood | NA | NA |
| PRJNA476781 | SRR7367623 | SLE | Blood | NA | NA |
| PRJNA476781 | SRR7367624 | SLE | Blood | NA | NA |
| PRJNA476781 | SRR7367625 | SLE | Blood | NA | NA |
| PRJNA476781 | SRR7367626 | SLE | Blood | NA | NA |
| PRJNA476781 | SRR7367627 | SLE | Blood | NA | NA |
| PRJNA476781 | SRR7367628 | SLE | Blood | NA | NA |
| PRJNA476781 | SRR7367629 | SLE | Blood | NA | NA |
| PRJNA476781 | SRR7367630 | SLE | Blood | NA | NA |
| PRJNA476781 | SRR7367631 | SLE | Blood | NA | NA |
| PRJNA476781 | SRR7367632 | SLE | Blood | NA | NA |
| PRJNA476781 | SRR7367633 | SLE | Blood | NA | NA |
| PRJNA476781 | SRR7367634 | SLE | Blood | NA | NA |
| PRJNA476781 | SRR7367635 | SLE | Blood | NA | NA |
| PRJNA476781 | SRR7367636 | SLE | Blood | NA | NA |
| PRJNA476781 | SRR7367637 | SLE | Blood | NA | NA |
| PRJNA476781 | SRR7367638 | SLE | Blood | NA | NA |
| PRJNA476781 | SRR7367639 | SLE | Blood | NA | NA |
| PRJNA476781 | SRR7367640 | SLE | Blood | NA | NA |
| PRJNA476781 | SRR7367641 | SLE | Blood | NA | NA |
| PRJNA476781 | SRR7367642 | SLE | Blood | NA | NA |
| PRJNA476781 | SRR7367643 | SLE | Blood | NA | NA |
| PRJNA476781 | SRR7367644 | SLE | Blood | NA | NA |
| PRJNA476781 | SRR7367645 | SLE | Blood | NA | NA |
| PRJNA476781 | SRR7367646 | SLE | Blood | NA | NA |
| PRJNA476781 | SRR7367647 | SLE | Blood | NA | NA |
| PRJNA476781 | SRR7367648 | SLE | Blood | NA | NA |
| PRJNA476781 | SRR7367649 | SLE | Blood | NA | NA |
| PRJNA476781 | SRR7367650 | SLE | Blood | NA | NA |
| PRJNA476781 | SRR7367651 | SLE | Blood | NA | NA |
| PRJNA476781 | SRR7367652 | SLE | Blood | NA | NA |
| PRJNA476781 | SRR7367653 | SLE | Blood | NA | NA |
| PRJNA476781 | SRR7367654 | SLE | Blood | NA | NA |
| PRJNA476781 | SRR7367655 | SLE | Blood | NA | NA |
| PRJNA476781 | SRR7367656 | SLE | Blood | NA | NA |
| PRJNA476781 | SRR7367657 | SLE | Blood | NA | NA |
| PRJNA476781 | SRR7367658 | SLE | Blood | NA | NA |
| PRJNA476781 | SRR7367659 | SLE | Blood | NA | NA |
| PRJNA476781 | SRR7367660 | SLE | Blood | NA | NA |
| PRJNA476781 | SRR7367661 | SLE | Blood | NA | NA |
| PRJNA476781 | SRR7367662 | SLE | Blood | NA | NA |
| PRJNA476781 | SRR7367663 | SLE | Blood | NA | NA |
| PRJNA476781 | SRR7367664 | SLE | Blood | NA | NA |
| PRJNA476781 | SRR7367665 | SLE | Blood | NA | NA |
| PRJNA476781 | SRR7367666 | SLE | Blood | NA | NA |
| PRJNA476781 | SRR7367667 | SLE | Blood | NA | NA |
| PRJNA476781 | SRR7367668 | SLE | Blood | NA | NA |
| PRJNA476781 | SRR7367669 | SLE | Blood | NA | NA |
| PRJNA476781 | SRR7367670 | SLE | Blood | NA | NA |
| PRJNA476781 | SRR7367671 | SLE | Blood | NA | NA |
| PRJNA476781 | SRR7367672 | SLE | Blood | NA | NA |
| PRJNA476781 | SRR7367673 | SLE | Blood | NA | NA |
| PRJNA476781 | SRR7367674 | SLE | Blood | NA | NA |
| PRJNA476781 | SRR7367675 | SLE | Blood | NA | NA |
| PRJNA476781 | SRR7367676 | SLE | Blood | NA | NA |
| PRJNA476781 | SRR7367677 | SLE | Blood | NA | NA |
| PRJNA476781 | SRR7367678 | SLE | Blood | NA | NA |
| PRJNA476781 | SRR7367679 | SLE | Blood | NA | NA |
| PRJNA476781 | SRR7367680 | SLE | Blood | NA | NA |
| PRJNA476781 | SRR7367681 | SLE | Blood | NA | NA |
| PRJNA476781 | SRR7367682 | SLE | Blood | NA | NA |
| PRJNA476781 | SRR7367683 | SLE | Blood | NA | NA |
| PRJNA476781 | SRR7367684 | SLE | Blood | NA | NA |
| PRJNA476781 | SRR7367685 | SLE | Blood | NA | NA |
| PRJNA476781 | SRR7367686 | SLE | Blood | NA | NA |
| PRJNA476781 | SRR7367687 | SLE | Blood | NA | NA |
| PRJNA476781 | SRR7367688 | SLE | Blood | NA | NA |
| PRJNA476781 | SRR7367689 | SLE | Blood | NA | NA |
| PRJNA476781 | SRR7367690 | SLE | Blood | NA | NA |
| PRJNA476781 | SRR7367691 | SLE | Blood | NA | NA |
| PRJNA476781 | SRR7367692 | SLE | Blood | NA | NA |
| PRJNA476781 | SRR7367693 | SLE | Blood | NA | NA |
| PRJNA476781 | SRR7367694 | SLE | Blood | NA | NA |
| PRJNA476781 | SRR7367695 | SLE | Blood | NA | NA |
| PRJNA476781 | SRR7367696 | SLE | Blood | NA | NA |
| PRJNA476781 | SRR7367697 | SLE | Blood | NA | NA |
| PRJNA476781 | SRR7367698 | SLE | Blood | NA | NA |
| PRJNA476781 | SRR7367699 | SLE | Blood | NA | NA |
| PRJNA476781 | SRR7367700 | SLE | Blood | NA | NA |
| PRJNA476781 | SRR7367701 | SLE | Blood | NA | NA |
| PRJNA476781 | SRR7367702 | SLE | Blood | NA | NA |
| PRJNA476781 | SRR7367703 | SLE | Blood | NA | NA |
| PRJNA476781 | SRR7367704 | SLE | Blood | NA | NA |
| PRJNA476781 | SRR7367705 | SLE | Blood | NA | NA |
| PRJNA476781 | SRR7367706 | SLE | Blood | NA | NA |
| PRJNA476781 | SRR7367707 | SLE | Blood | NA | NA |
| PRJNA476781 | SRR7367708 | SLE | Blood | NA | NA |
| PRJNA476781 | SRR7367709 | SLE | Blood | NA | NA |
| PRJNA476781 | SRR7367710 | SLE | Blood | NA | NA |
| PRJNA476781 | SRR7367711 | SLE | Blood | NA | NA |
| PRJNA476781 | SRR7367712 | SLE | Blood | NA | NA |
| PRJNA476781 | SRR7367713 | SLE | Blood | NA | NA |
| PRJNA476781 | SRR7367714 | SLE | Blood | NA | NA |
| PRJNA476781 | SRR7367715 | SLE | Blood | NA | NA |
| PRJNA476781 | SRR7367716 | SLE | Blood | NA | NA |
| PRJNA476781 | SRR7367717 | SLE | Blood | NA | NA |
| PRJNA476781 | SRR7367718 | SLE | Blood | NA | NA |
| PRJNA476781 | SRR7367719 | SLE | Blood | NA | NA |
| PRJNA476781 | SRR7367720 | SLE | Blood | NA | NA |
| PRJNA476781 | SRR7367721 | SLE | Blood | NA | NA |
| PRJNA476781 | SRR7367722 | SLE | Blood | NA | NA |
| PRJNA476781 | SRR7367723 | SLE | Blood | NA | NA |
| PRJNA476781 | SRR7367724 | SLE | Blood | NA | NA |
| PRJNA476781 | SRR7367725 | SLE | Blood | NA | NA |
| PRJNA476781 | SRR7367726 | SLE | Blood | NA | NA |
| PRJNA476781 | SRR7367727 | SLE | Blood | NA | NA |
| PRJNA476781 | SRR7367728 | SLE | Blood | NA | NA |
| PRJNA476781 | SRR7367729 | SLE | Blood | NA | NA |
| PRJNA476781 | SRR7367730 | SLE | Blood | NA | NA |
| PRJNA476781 | SRR7367731 | SLE | Blood | NA | NA |
| PRJNA476781 | SRR7367732 | SLE | Blood | NA | NA |
| PRJNA476781 | SRR7367733 | SLE | Blood | NA | NA |
| PRJNA476781 | SRR7367734 | SLE | Blood | NA | NA |
| PRJNA476781 | SRR7367735 | SLE | Blood | NA | NA |
| PRJNA476781 | SRR7367736 | SLE | Blood | NA | NA |
| PRJNA476781 | SRR7367737 | SLE | Blood | NA | NA |
| PRJNA476781 | SRR7367738 | SLE | Blood | NA | NA |
| PRJNA476781 | SRR7367739 | SLE | Blood | NA | NA |
| PRJNA476781 | SRR7367740 | SLE | Blood | NA | NA |
| PRJNA476781 | SRR7367741 | SLE | Blood | NA | NA |
| PRJNA476781 | SRR7367742 | SLE | Blood | NA | NA |
| PRJNA476781 | SRR7367743 | SLE | Blood | NA | NA |
| PRJNA476781 | SRR7367744 | SLE | Blood | NA | NA |
| PRJNA476781 | SRR7367745 | SLE | Blood | NA | NA |
| PRJNA476781 | SRR7367746 | SLE | Blood | NA | NA |
| PRJNA476781 | SRR7367747 | SLE | Blood | NA | NA |
| PRJNA476781 | SRR7367748 | SLE | Blood | NA | NA |
| PRJNA476781 | SRR7367749 | SLE | Blood | NA | NA |
| PRJNA476781 | SRR7367750 | SLE | Blood | NA | NA |
| PRJNA476781 | SRR7367751 | SLE | Blood | NA | NA |
| PRJNA476781 | SRR7367752 | SLE | Blood | NA | NA |
| PRJNA476781 | SRR7367753 | SLE | Blood | NA | NA |
| PRJNA476781 | SRR7367754 | SLE | Blood | NA | NA |
| PRJNA476781 | SRR7367755 | SLE | Blood | NA | NA |
| PRJNA476781 | SRR7367756 | SLE | Blood | NA | NA |
| PRJNA476781 | SRR7367757 | SLE | Blood | NA | NA |
| PRJNA476781 | SRR7367758 | SLE | Blood | NA | NA |
| PRJNA476781 | SRR7367759 | SLE | Blood | NA | NA |
| PRJNA476781 | SRR7367760 | SLE | Blood | NA | NA |
| PRJNA476781 | SRR7367761 | SLE | Blood | NA | NA |
| PRJNA476781 | SRR7367762 | SLE | Blood | NA | NA |
| PRJNA476781 | SRR7367763 | SLE | Blood | NA | NA |
| PRJNA476781 | SRR7367764 | SLE | Blood | NA | NA |
| PRJNA476781 | SRR7367765 | SLE | Blood | NA | NA |
| PRJNA476781 | SRR7367766 | SLE | Blood | NA | NA |
| PRJNA476781 | SRR7367767 | SLE | Blood | NA | NA |
| PRJNA476781 | SRR7367768 | SLE | Blood | NA | NA |
| PRJNA476781 | SRR7367769 | SLE | Blood | NA | NA |
| PRJNA476781 | SRR7367770 | SLE | Blood | NA | NA |
| PRJNA476781 | SRR7367771 | SLE | Blood | NA | NA |
| PRJNA476781 | SRR7367772 | SLE | Blood | NA | NA |
| PRJNA476781 | SRR7367773 | SLE | Blood | NA | NA |
| PRJNA476781 | SRR7367774 | SLE | Blood | NA | NA |
| PRJNA476781 | SRR7367775 | SLE | Blood | NA | NA |
| PRJNA476781 | SRR7367776 | SLE | Blood | NA | NA |
| PRJNA476781 | SRR7367777 | SLE | Blood | NA | NA |
| PRJNA476781 | SRR7367778 | SLE | Blood | NA | NA |
| PRJNA476781 | SRR7367779 | SLE | Blood | NA | NA |
| PRJNA476781 | SRR7367780 | SLE | Blood | NA | NA |
| PRJNA476781 | SRR7367781 | SLE | Blood | NA | NA |
| PRJNA476781 | SRR7367782 | SLE | Blood | NA | NA |
| PRJNA476781 | SRR7367783 | SLE | Blood | NA | NA |
| PRJNA476781 | SRR7367784 | SLE | Blood | NA | NA |
| PRJNA476781 | SRR7367785 | SLE | Blood | NA | NA |
| PRJNA476781 | SRR7367786 | SLE | Blood | NA | NA |
| PRJNA476781 | SRR7367787 | SLE | Blood | NA | NA |
| PRJNA476781 | SRR7367788 | SLE | Blood | NA | NA |
| PRJNA476781 | SRR7367789 | SLE | Blood | NA | NA |
| PRJNA476781 | SRR7367790 | SLE | Blood | NA | NA |
| PRJNA476781 | SRR7367791 | SLE | Blood | NA | NA |
| PRJNA476781 | SRR7367792 | SLE | Blood | NA | NA |
| PRJNA476781 | SRR7367793 | SLE | Blood | NA | NA |
| PRJNA476781 | SRR7367794 | SLE | Blood | NA | NA |
| PRJNA476781 | SRR7367795 | SLE | Blood | NA | NA |
| PRJNA476781 | SRR7367796 | SLE | Blood | NA | NA |
| PRJNA476781 | SRR7367797 | SLE | Blood | NA | NA |
| PRJNA476781 | SRR7367798 | SLE | Blood | NA | NA |
| PRJNA476781 | SRR7367799 | SLE | Blood | NA | NA |
| PRJNA476781 | SRR7367800 | SLE | Blood | NA | NA |
| PRJNA476781 | SRR7367801 | SLE | Blood | NA | NA |
| PRJNA476781 | SRR7367802 | SLE | Blood | NA | NA |
| PRJNA476781 | SRR7367803 | SLE | Blood | NA | NA |
| PRJNA476781 | SRR7367804 | SLE | Blood | NA | NA |
| PRJNA476781 | SRR7367805 | SLE | Blood | NA | NA |
| PRJNA476781 | SRR7367806 | SLE | Blood | NA | NA |
| PRJNA476781 | SRR7367807 | SLE | Blood | NA | NA |
| PRJNA476781 | SRR7367808 | SLE | Blood | NA | NA |
| PRJNA476781 | SRR7367809 | SLE | Blood | NA | NA |
| PRJNA476781 | SRR7367810 | SLE | Blood | NA | NA |
| PRJNA476781 | SRR7367811 | SLE | Blood | NA | NA |
| PRJNA476781 | SRR7367812 | SLE | Blood | NA | NA |
| PRJNA476781 | SRR7367813 | SLE | Blood | NA | NA |
| PRJNA476781 | SRR7367814 | SLE | Blood | NA | NA |
| PRJNA476781 | SRR7367815 | SLE | Blood | NA | NA |
| PRJNA476781 | SRR7367816 | SLE | Blood | NA | NA |
| PRJNA476781 | SRR7367817 | SLE | Blood | NA | NA |
| PRJNA476781 | SRR7367818 | SLE | Blood | NA | NA |
| PRJNA476781 | SRR7367819 | SLE | Blood | NA | NA |
| PRJNA476781 | SRR7367820 | SLE | Blood | NA | NA |
| PRJNA476781 | SRR7367821 | SLE | Blood | NA | NA |
| PRJNA476781 | SRR7367822 | SLE | Blood | NA | NA |
| PRJNA476781 | SRR7367823 | SLE | Blood | NA | NA |
| PRJNA476781 | SRR7367824 | SLE | Blood | NA | NA |
| PRJNA476781 | SRR7367825 | SLE | Blood | NA | NA |
| PRJNA476781 | SRR7367826 | SLE | Blood | NA | NA |
| PRJNA476781 | SRR7367827 | SLE | Blood | NA | NA |
| PRJNA476781 | SRR7367828 | SLE | Blood | NA | NA |
| PRJNA476781 | SRR7367829 | SLE | Blood | NA | NA |
| PRJNA476781 | SRR7367830 | SLE | Blood | NA | NA |
| PRJNA476781 | SRR7367831 | SLE | Blood | NA | NA |
| PRJNA476781 | SRR7367832 | SLE | Blood | NA | NA |
| PRJNA476781 | SRR7367833 | SLE | Blood | NA | NA |
| PRJNA476781 | SRR7367834 | SLE | Blood | NA | NA |
| PRJNA476781 | SRR7367835 | SLE | Blood | NA | NA |
| PRJNA476781 | SRR7367836 | SLE | Blood | NA | NA |
| PRJNA476781 | SRR7367837 | SLE | Blood | NA | NA |
| PRJNA476781 | SRR7367838 | SLE | Blood | NA | NA |
| PRJNA476781 | SRR7367839 | SLE | Blood | NA | NA |
| PRJNA476781 | SRR7367840 | SLE | Blood | NA | NA |
| PRJNA476781 | SRR7367841 | SLE | Blood | NA | NA |
| PRJNA476781 | SRR7367842 | SLE | Blood | NA | NA |
| PRJNA476781 | SRR7367843 | SLE | Blood | NA | NA |
| PRJNA476781 | SRR7367844 | SLE | Blood | NA | NA |
| PRJNA476781 | SRR7367845 | SLE | Blood | NA | NA |
| PRJNA476781 | SRR7367846 | SLE | Blood | NA | NA |
| PRJNA476781 | SRR7367847 | SLE | Blood | NA | NA |
| PRJNA476781 | SRR7367848 | SLE | Blood | NA | NA |
| PRJNA476781 | SRR7367849 | SLE | Blood | NA | NA |
| PRJNA476781 | SRR7367850 | SLE | Blood | NA | NA |
| PRJNA476781 | SRR7367851 | SLE | Blood | NA | NA |
| PRJNA476781 | SRR7367852 | SLE | Blood | NA | NA |
| PRJNA476781 | SRR7367853 | SLE | Blood | NA | NA |
| PRJNA476781 | SRR7367854 | SLE | Blood | NA | NA |
| PRJNA476781 | SRR7367855 | SLE | Blood | NA | NA |
| PRJNA476781 | SRR7367856 | SLE | Blood | NA | NA |
| PRJNA476781 | SRR7367857 | SLE | Blood | NA | NA |
| PRJNA476781 | SRR7367858 | SLE | Blood | NA | NA |
| PRJNA476781 | SRR7367859 | SLE | Blood | NA | NA |
| PRJNA476781 | SRR7367860 | SLE | Blood | NA | NA |
| PRJNA476781 | SRR7367861 | SLE | Blood | NA | NA |
| PRJNA476781 | SRR7367862 | SLE | Blood | NA | NA |
| PRJNA476781 | SRR7367863 | SLE | Blood | NA | NA |
| PRJNA476781 | SRR7367864 | SLE | Blood | NA | NA |
| PRJNA476781 | SRR7367865 | SLE | Blood | NA | NA |
| PRJNA476781 | SRR7367866 | SLE | Blood | NA | NA |
| PRJNA476781 | SRR7367867 | SLE | Blood | NA | NA |
| PRJNA476781 | SRR7367868 | SLE | Blood | NA | NA |
| PRJNA476781 | SRR7367869 | SLE | Blood | NA | NA |
| PRJNA476781 | SRR7367870 | SLE | Blood | NA | NA |
| PRJNA476781 | SRR7367871 | SLE | Blood | NA | NA |
| PRJNA476781 | SRR7367872 | SLE | Blood | NA | NA |
| PRJNA476781 | SRR7367873 | SLE | Blood | NA | NA |
| PRJNA476781 | SRR7367874 | SLE | Blood | NA | NA |
| PRJNA476781 | SRR7367875 | SLE | Blood | NA | NA |
| PRJNA476781 | SRR7367876 | SLE | Blood | NA | NA |
| PRJNA476781 | SRR7367877 | SLE | Blood | NA | NA |
| PRJNA476781 | SRR7367878 | SLE | Blood | NA | NA |
| PRJNA476781 | SRR7367879 | SLE | Blood | NA | NA |
| PRJNA476781 | SRR7367880 | SLE | Blood | NA | NA |
| PRJNA476781 | SRR7367881 | SLE | Blood | NA | NA |
| PRJNA476781 | SRR7367882 | SLE | Blood | NA | NA |
| PRJNA476781 | SRR7367883 | SLE | Blood | NA | NA |
| PRJNA476781 | SRR7367884 | SLE | Blood | NA | NA |
| PRJNA476781 | SRR7367885 | SLE | Blood | NA | NA |
| PRJNA476781 | SRR7367886 | SLE | Blood | NA | NA |
| PRJNA476781 | SRR7367887 | SLE | Blood | NA | NA |
| PRJNA476781 | SRR7367888 | SLE | Blood | NA | NA |
| PRJNA476781 | SRR7367889 | SLE | Blood | NA | NA |
| PRJNA476781 | SRR7367890 | SLE | Blood | NA | NA |
| PRJNA476781 | SRR7367891 | SLE | Blood | NA | NA |
| PRJNA476781 | SRR7367892 | SLE | Blood | NA | NA |
| PRJNA476781 | SRR7367893 | SLE | Blood | NA | NA |
| PRJNA476781 | SRR7367894 | SLE | Blood | NA | NA |
| PRJNA476781 | SRR7367895 | SLE | Blood | NA | NA |
| PRJNA476781 | SRR7367896 | SLE | Blood | NA | NA |
| PRJNA476781 | SRR7367897 | SLE | Blood | NA | NA |
| PRJNA476781 | SRR7367898 | SLE | Blood | NA | NA |
| PRJNA476781 | SRR7367899 | SLE | Blood | NA | NA |
| PRJNA476781 | SRR7367900 | SLE | Blood | NA | NA |
| PRJNA476781 | SRR7367901 | SLE | Blood | NA | NA |
| PRJNA476781 | SRR7367902 | SLE | Blood | NA | NA |
| PRJNA476781 | SRR7367903 | SLE | Blood | NA | NA |
| PRJNA476781 | SRR7367904 | SLE | Blood | NA | NA |
| PRJNA476781 | SRR7367905 | SLE | Blood | NA | NA |
| PRJNA476781 | SRR7367906 | SLE | Blood | NA | NA |
| PRJNA476781 | SRR7367907 | SLE | Blood | NA | NA |
| PRJNA476781 | SRR7367908 | SLE | Blood | NA | NA |
| PRJNA476781 | SRR7367909 | SLE | Blood | NA | NA |
| PRJNA476781 | SRR7367910 | SLE | Blood | NA | NA |
| PRJNA476781 | SRR7367911 | SLE | Blood | NA | NA |
| PRJNA476781 | SRR7367912 | SLE | Blood | NA | NA |
| PRJNA476781 | SRR7367913 | SLE | Blood | NA | NA |
| PRJNA476781 | SRR7367914 | SLE | Blood | NA | NA |
| PRJNA476781 | SRR7367915 | SLE | Blood | NA | NA |
| PRJNA476781 | SRR7367916 | SLE | Blood | NA | NA |
| PRJNA476781 | SRR7367917 | SLE | Blood | NA | NA |
| PRJNA476781 | SRR7367918 | SLE | Blood | NA | NA |
| PRJNA476781 | SRR7367919 | SLE | Blood | NA | NA |
| PRJNA476781 | SRR7367920 | SLE | Blood | NA | NA |
| PRJNA476781 | SRR7367921 | SLE | Blood | NA | NA |
| PRJNA476781 | SRR7367922 | SLE | Blood | NA | NA |
| PRJNA476781 | SRR7367923 | SLE | Blood | NA | NA |
| PRJNA476781 | SRR7367924 | SLE | Blood | NA | NA |
| PRJNA476781 | SRR7367925 | SLE | Blood | NA | NA |
| PRJNA476781 | SRR7367926 | SLE | Blood | NA | NA |
| PRJNA476781 | SRR7367927 | SLE | Blood | NA | NA |
| PRJNA476781 | SRR7367928 | SLE | Blood | NA | NA |
| PRJNA476781 | SRR7367929 | SLE | Blood | NA | NA |
| PRJNA476781 | SRR7367930 | SLE | Blood | NA | NA |
| PRJNA476781 | SRR7367931 | SLE | Blood | NA | NA |
| PRJNA476781 | SRR7367932 | SLE | Blood | NA | NA |
| PRJNA476781 | SRR7367933 | SLE | Blood | NA | NA |
| PRJNA476781 | SRR7367934 | SLE | Blood | NA | NA |
| PRJNA476781 | SRR7367935 | SLE | Blood | NA | NA |
| PRJNA476781 | SRR7367936 | SLE | Blood | NA | NA |
| PRJNA476781 | SRR7367937 | SLE | Blood | NA | NA |
| PRJNA476781 | SRR7367938 | SLE | Blood | NA | NA |
| PRJNA476781 | SRR7367939 | SLE | Blood | NA | NA |
| PRJNA476781 | SRR7367940 | SLE | Blood | NA | NA |
| PRJNA476781 | SRR7367941 | SLE | Blood | NA | NA |
| PRJNA476781 | SRR7367942 | SLE | Blood | NA | NA |
| PRJNA476781 | SRR7367943 | SLE | Blood | NA | NA |
| PRJNA476781 | SRR7367944 | SLE | Blood | NA | NA |
| PRJNA476781 | SRR7367945 | SLE | Blood | NA | NA |
| PRJNA476781 | SRR7367946 | SLE | Blood | NA | NA |
| PRJNA476781 | SRR7367947 | SLE | Blood | NA | NA |
| PRJNA476781 | SRR7367948 | SLE | Blood | NA | NA |
| PRJNA476781 | SRR7367949 | SLE | Blood | NA | NA |
| PRJNA476781 | SRR7367950 | SLE | Blood | NA | NA |
| PRJNA476781 | SRR7367951 | SLE | Blood | NA | NA |
| PRJNA476781 | SRR7367952 | SLE | Blood | NA | NA |
| PRJNA476781 | SRR7367953 | SLE | Blood | NA | NA |
| PRJNA476781 | SRR7367954 | SLE | Blood | NA | NA |
| PRJNA476781 | SRR7367955 | SLE | Blood | NA | NA |
| PRJNA476781 | SRR7367956 | SLE | Blood | NA | NA |
| PRJNA476781 | SRR7367957 | SLE | Blood | NA | NA |
| PRJNA476781 | SRR7367958 | SLE | Blood | NA | NA |
| PRJNA476781 | SRR7367959 | SLE | Blood | NA | NA |
| PRJNA476781 | SRR7367960 | SLE | Blood | NA | NA |
| PRJNA476781 | SRR7367961 | SLE | Blood | NA | NA |
| PRJNA476781 | SRR7367962 | SLE | Blood | NA | NA |
| PRJNA476781 | SRR7367963 | SLE | Blood | NA | NA |
| PRJNA476781 | SRR7367964 | SLE | Blood | NA | NA |
| PRJNA476781 | SRR7367965 | SLE | Blood | NA | NA |
| PRJNA476781 | SRR7367966 | SLE | Blood | NA | NA |
| PRJNA476781 | SRR7367967 | SLE | Blood | NA | NA |
| PRJNA476781 | SRR7367968 | SLE | Blood | NA | NA |
| PRJNA476781 | SRR7367969 | SLE | Blood | NA | NA |
| PRJNA476781 | SRR7367970 | SLE | Blood | NA | NA |
| PRJNA476781 | SRR7367971 | SLE | Blood | NA | NA |
| PRJNA476781 | SRR7367972 | SLE | Blood | NA | NA |
| PRJNA476781 | SRR7367973 | SLE | Blood | NA | NA |
| PRJNA476781 | SRR7367974 | SLE | Blood | NA | NA |
| PRJNA476781 | SRR7367975 | SLE | Blood | NA | NA |
| PRJNA476781 | SRR7367976 | SLE | Blood | NA | NA |
| PRJNA476781 | SRR7367977 | SLE | Blood | NA | NA |
| PRJNA476781 | SRR7367978 | SLE | Blood | NA | NA |
| PRJNA476781 | SRR7367979 | SLE | Blood | NA | NA |
| PRJNA476781 | SRR7367980 | SLE | Blood | NA | NA |
| PRJNA476781 | SRR7367981 | SLE | Blood | NA | NA |
| PRJNA476781 | SRR7367982 | SLE | Blood | NA | NA |
| PRJNA476781 | SRR7367983 | SLE | Blood | NA | NA |
| PRJNA476781 | SRR7367984 | SLE | Blood | NA | NA |
| PRJNA476781 | SRR7367985 | SLE | Blood | NA | NA |
| PRJNA476781 | SRR7367986 | SLE | Blood | NA | NA |
| PRJNA476781 | SRR7367987 | SLE | Blood | NA | NA |
| PRJNA476781 | SRR7367988 | SLE | Blood | NA | NA |
| PRJNA476781 | SRR7367989 | SLE | Blood | NA | NA |
| PRJNA476781 | SRR7367990 | SLE | Blood | NA | NA |
| PRJNA476781 | SRR7367991 | SLE | Blood | NA | NA |
| PRJNA476781 | SRR7367992 | SLE | Blood | NA | NA |
| PRJNA476781 | SRR7367993 | SLE | Blood | NA | NA |
| PRJNA476781 | SRR7367994 | SLE | Blood | NA | NA |
| PRJNA476781 | SRR7367995 | SLE | Blood | NA | NA |
| PRJNA476781 | SRR7367996 | SLE | Blood | NA | NA |
| PRJNA476781 | SRR7367997 | SLE | Blood | NA | NA |
| PRJNA476781 | SRR7367998 | SLE | Blood | NA | NA |
| PRJNA476781 | SRR7367999 | SLE | Blood | NA | NA |
| PRJNA476781 | SRR7368000 | SLE | Blood | NA | NA |
| PRJNA476781 | SRR7368001 | SLE | Blood | NA | NA |
| PRJNA476781 | SRR7368002 | SLE | Blood | NA | NA |
| PRJNA476781 | SRR7368003 | SLE | Blood | NA | NA |
| PRJNA476781 | SRR7368004 | SLE | Blood | NA | NA |
| PRJNA476781 | SRR7368005 | SLE | Blood | NA | NA |
| PRJNA476781 | SRR7368006 | SLE | Blood | NA | NA |
| PRJNA476781 | SRR7368007 | SLE | Blood | NA | NA |
| PRJNA476781 | SRR7368008 | SLE | Blood | NA | NA |
| PRJNA476781 | SRR7368009 | SLE | Blood | NA | NA |
| PRJNA476781 | SRR7368010 | SLE | Blood | NA | NA |
| PRJNA476781 | SRR7368011 | SLE | Blood | NA | NA |
| PRJNA476781 | SRR7368012 | SLE | Blood | NA | NA |
| PRJNA476781 | SRR7368013 | SLE | Blood | NA | NA |
| PRJNA476781 | SRR7368014 | SLE | Blood | NA | NA |
| PRJNA476781 | SRR7368015 | SLE | Blood | NA | NA |
| PRJNA476781 | SRR7368016 | SLE | Blood | NA | NA |
| PRJNA476781 | SRR7368017 | SLE | Blood | NA | NA |
| PRJNA476781 | SRR7368018 | SLE | Blood | NA | NA |
| PRJNA476781 | SRR7368019 | SLE | Blood | NA | NA |
| PRJNA476781 | SRR7368020 | SLE | Blood | NA | NA |
| PRJNA476781 | SRR7368021 | SLE | Blood | NA | NA |
| PRJNA476781 | SRR7368022 | SLE | Blood | NA | NA |
| PRJNA476781 | SRR7368023 | SLE | Blood | NA | NA |
| PRJNA476781 | SRR7368024 | SLE | Blood | NA | NA |
| PRJNA476781 | SRR7368025 | SLE | Blood | NA | NA |
| PRJNA476781 | SRR7368026 | SLE | Blood | NA | NA |
| PRJNA476781 | SRR7368027 | SLE | Blood | NA | NA |
| PRJNA476781 | SRR7368028 | SLE | Blood | NA | NA |
| PRJNA476781 | SRR7368029 | SLE | Blood | NA | NA |
| PRJNA476781 | SRR7368030 | SLE | Blood | NA | NA |
| PRJNA476781 | SRR7368031 | SLE | Blood | NA | NA |
| PRJNA476781 | SRR7368032 | SLE | Blood | NA | NA |
| PRJNA476781 | SRR7368033 | SLE | Blood | NA | NA |
| PRJNA476781 | SRR7368034 | SLE | Blood | NA | NA |
| PRJNA476781 | SRR7368035 | SLE | Blood | NA | NA |
| PRJNA476781 | SRR7368036 | SLE | Blood | NA | NA |
| PRJNA476781 | SRR7368037 | SLE | Blood | NA | NA |
| PRJNA476781 | SRR7368038 | SLE | Blood | NA | NA |
| PRJNA476781 | SRR7368039 | SLE | Blood | NA | NA |
| PRJNA476781 | SRR7368040 | SLE | Blood | NA | NA |
| PRJNA476781 | SRR7368041 | SLE | Blood | NA | NA |
| PRJNA476781 | SRR7368042 | SLE | Blood | NA | NA |
| PRJNA476781 | SRR7368043 | SLE | Blood | NA | NA |
| PRJNA476781 | SRR7368044 | SLE | Blood | NA | NA |
| PRJNA476781 | SRR7368045 | SLE | Blood | NA | NA |
| PRJNA476781 | SRR7368046 | SLE | Blood | NA | NA |
| PRJNA476781 | SRR7368047 | SLE | Blood | NA | NA |
| PRJNA476781 | SRR7368048 | SLE | Blood | NA | NA |
| PRJNA476781 | SRR7368049 | SLE | Blood | NA | NA |
| PRJNA476781 | SRR7368050 | SLE | Blood | NA | NA |
| PRJNA476781 | SRR7368051 | SLE | Blood | NA | NA |
| PRJNA476781 | SRR7368052 | SLE | Blood | NA | NA |
| PRJNA476781 | SRR7368053 | SLE | Blood | NA | NA |
| PRJNA476781 | SRR7368054 | SLE | Blood | NA | NA |
| PRJNA476781 | SRR7368055 | SLE | Blood | NA | NA |
| PRJNA476781 | SRR7368056 | SLE | Blood | NA | NA |
| PRJNA476781 | SRR7368057 | SLE | Blood | NA | NA |
| PRJNA476781 | SRR7368058 | SLE | Blood | NA | NA |
| PRJNA476781 | SRR7368059 | SLE | Blood | NA | NA |
| PRJNA476781 | SRR7368060 | SLE | Blood | NA | NA |
| PRJNA476781 | SRR7368061 | SLE | Blood | NA | NA |
| PRJNA476781 | SRR7368062 | SLE | Blood | NA | NA |
| PRJNA476781 | SRR7368063 | SLE | Blood | NA | NA |
| PRJNA476781 | SRR7368064 | SLE | Blood | NA | NA |
| PRJNA476781 | SRR7368065 | SLE | Blood | NA | NA |
| PRJNA476781 | SRR7368066 | SLE | Blood | NA | NA |
| PRJNA476781 | SRR7368067 | SLE | Blood | NA | NA |
| PRJNA476781 | SRR7368068 | SLE | Blood | NA | NA |
| PRJNA476781 | SRR7368069 | SLE | Blood | NA | NA |
| PRJNA483394 | SRR7618095 | SLE | Blood | 71 | NA |
| PRJNA483394 | SRR7618096 | SLE | Blood | 62 | NA |
| PRJNA483394 | SRR7618097 | SLE | Blood | 71 | NA |
| PRJNA483394 | SRR7618098 | SLE | Blood | 51 | NA |
| PRJNA483394 | SRR7618099 | SLE | Blood | 43 | NA |
| PRJNA483394 | SRR7618100 | SLE | Blood | 34 | NA |
| PRJNA483394 | SRR7618101 | SLE | Blood | 56 | NA |
| PRJNA483394 | SRR7618102 | SLE | Blood | 39 | NA |
| PRJNA483394 | SRR7618103 | SLE | Blood | 52 | NA |
| PRJNA483394 | SRR7618104 | Control | Blood | 40 | NA |
| PRJNA483394 | SRR7618105 | SLE | Blood | 34 | NA |
| PRJNA483394 | SRR7618106 | SLE | Blood | 21 | NA |
| PRJNA483394 | SRR7618107 | SLE | Blood | 28 | NA |
| PRJNA483394 | SRR7618108 | SLE | Blood | 42 | NA |
| PRJNA483394 | SRR7618109 | SLE | Blood | 59 | NA |
| PRJNA483394 | SRR7618110 | SLE | Blood | 50 | NA |
| PRJNA483394 | SRR7618111 | SLE | Blood | 36 | NA |
| PRJNA483394 | SRR7618112 | SLE | Blood | 53 | NA |
| PRJNA483394 | SRR7618113 | Control | Blood | 35 | NA |
| PRJNA483394 | SRR7618114 | Control | Blood | 50 | NA |
| PRJNA483394 | SRR7618115 | Control | Blood | 34 | NA |
| PRJNA483394 | SRR7618116 | Control | Blood | 44 | NA |
| PRJNA483394 | SRR7618117 | Control | Blood | 33 | NA |
| PRJNA483394 | SRR7618358 | SLE | Blood | 32 | NA |
| PRJNA496570 | SRR8074216 | Control | Blood | NA | NA |
| PRJNA496570 | SRR8074217 | Control | Blood | NA | NA |
| PRJNA496570 | SRR8074218 | Control | Blood | NA | NA |
| PRJNA496570 | SRR8074219 | Control | Blood | NA | NA |
| PRJNA496570 | SRR8074220 | SLE | Blood | NA | NA |
| PRJNA496570 | SRR8074221 | SLE | Blood | NA | NA |
| PRJNA496570 | SRR8074222 | SLE | Blood | NA | NA |
| PRJNA496570 | SRR8074223 | SLE | Blood | NA | NA |
| PRJNA496570 | SRR8074224 | SLE | Blood | NA | NA |
| PRJNA496570 | SRR8074225 | SLE | Blood | NA | NA |
| PRJNA496570 | SRR8074226 | SLE | Blood | NA | NA |
| PRJNA496570 | SRR8074227 | SLE | Blood | NA | NA |
| PRJNA505280 | SRR8181357 | SLE | Blood | NA | NA |
| PRJNA505280 | SRR8181358 | SLE | Blood | NA | NA |
| PRJNA505280 | SRR8181359 | SLE | Blood | NA | NA |
| PRJNA505280 | SRR8181360 | SLE | Blood | NA | NA |
| PRJNA505280 | SRR8181361 | SLE | Blood | NA | NA |
| PRJNA505280 | SRR8181362 | SLE | Blood | NA | NA |
| PRJNA505280 | SRR8181363 | SLE | Blood | NA | NA |
| PRJNA505280 | SRR8181364 | SLE | Blood | NA | NA |
| PRJNA505280 | SRR8181365 | SLE | Blood | NA | NA |
| PRJNA505280 | SRR8181366 | SLE | Blood | NA | NA |
| PRJNA505280 | SRR8181367 | SLE | Blood | NA | NA |
| PRJNA505280 | SRR8181368 | SLE | Blood | NA | NA |
| PRJNA505280 | SRR8181369 | SLE | Blood | NA | NA |
| PRJNA505280 | SRR8181370 | SLE | Blood | NA | NA |
| PRJNA505280 | SRR8181371 | SLE | Blood | NA | NA |
| PRJNA505280 | SRR8181372 | SLE | Blood | NA | NA |
| PRJNA505280 | SRR8181373 | SLE | Blood | NA | NA |
| PRJNA505280 | SRR8181374 | SLE | Blood | NA | NA |
| PRJNA505280 | SRR8181375 | SLE | Blood | NA | NA |
| PRJNA505280 | SRR8181376 | SLE | Blood | NA | NA |
| PRJNA505280 | SRR8181377 | Control | Blood | NA | NA |
| PRJNA505280 | SRR8181378 | Control | Blood | NA | NA |
| PRJNA505280 | SRR8181379 | Control | Blood | NA | NA |
| PRJNA505280 | SRR8181380 | Control | Blood | NA | NA |
| PRJNA505280 | SRR8181381 | Control | Blood | NA | NA |
| PRJNA505280 | SRR8181382 | Control | Blood | NA | NA |
| PRJNA579362 | SRR10342368 | SLE | Blood | NA | NA |
| PRJNA579362 | SRR10342369 | SLE | Blood | NA | NA |
| PRJNA579362 | SRR10342370 | Control | Blood | NA | NA |
| PRJNA579362 | SRR10342371 | Control | Blood | NA | NA |
| PRJNA645252 | SRR12190417 | Control | Blood | NA | NA |
| PRJNA645252 | SRR12190418 | Control | Blood | NA | NA |
| PRJNA645252 | SRR12190419 | Control | Blood | NA | NA |
| PRJNA645252 | SRR12190420 | Control | Blood | NA | NA |
| PRJNA645252 | SRR12190421 | Control | Blood | NA | NA |
| PRJNA645252 | SRR12190422 | Control | Blood | NA | NA |
| PRJNA645252 | SRR12190423 | Control | Blood | NA | NA |
| PRJNA645252 | SRR12190424 | SLE | Blood | NA | NA |
| PRJNA645252 | SRR12190425 | SLE | Blood | NA | NA |
| PRJNA645252 | SRR12190426 | SLE | Blood | NA | NA |
| PRJNA645252 | SRR12190427 | SLE | Blood | NA | NA |
| PRJNA645252 | SRR12190428 | SLE | Blood | NA | NA |
| PRJNA645252 | SRR12190429 | Control | Blood | NA | NA |
| PRJNA645252 | SRR12190430 | SLE | Blood | NA | NA |
| PRJNA645252 | SRR12190431 | SLE | Blood | NA | NA |
| PRJNA645252 | SRR12190432 | SLE | Blood | NA | NA |
| PRJNA645252 | SRR12190433 | SLE | Blood | NA | NA |
| PRJNA645252 | SRR12190434 | SLE | Blood | NA | NA |
| PRJNA645252 | SRR12190435 | SLE | Blood | NA | NA |
| PRJNA645252 | SRR12190436 | SLE | Blood | NA | NA |
| PRJNA645252 | SRR12190437 | SLE | Blood | NA | NA |
| PRJNA645252 | SRR12190438 | Control | Blood | NA | NA |
| PRJNA645252 | SRR12190439 | Control | Blood | NA | NA |
| PRJNA645252 | SRR12190440 | Control | Blood | NA | NA |
| PRJNA645252 | SRR12190441 | Control | Blood | NA | NA |
| PRJNA667695 | SRR12780584 | Control | Blood | NA | female |
| PRJNA667695 | SRR12780585 | Control | Blood | NA | male |
| PRJNA667695 | SRR12780586 | Control | Blood | NA | female |
| PRJNA667695 | SRR12780587 | Control | Blood | NA | female |
| PRJNA667695 | SRR12780588 | Control | Blood | NA | female |
| PRJNA667695 | SRR12780589 | Control | Blood | NA | male |
| PRJNA667695 | SRR12780590 | Control | Blood | NA | female |
| PRJNA667695 | SRR12780591 | Control | Blood | NA | female |
| PRJNA667695 | SRR12780592 | Control | Blood | NA | female |
| PRJNA667695 | SRR12780593 | Control | Blood | NA | male |
| PRJNA667695 | SRR12780594 | Control | Blood | NA | female |
| PRJNA667695 | SRR12780595 | Control | Blood | NA | female |
| PRJNA667695 | SRR12780596 | Control | Blood | NA | female |
| PRJNA667695 | SRR12780597 | Control | Blood | NA | male |
| PRJNA667695 | SRR12780598 | Control | Blood | NA | female |
| PRJNA667695 | SRR12780599 | Control | Blood | NA | female |
| PRJNA683207 | SRR13214141 | Control | Blood | NA | NA |
| PRJNA683207 | SRR13214142 | Control | Blood | NA | NA |
| PRJNA683207 | SRR13214143 | Control | Blood | NA | NA |
| PRJNA683207 | SRR13214144 | Control | Blood | NA | NA |
| PRJNA683207 | SRR13214145 | Control | Blood | NA | NA |
| PRJNA683207 | SRR13214146 | SLE | Blood | NA | NA |
| PRJNA683207 | SRR13214147 | SLE | Blood | NA | NA |
| PRJNA683207 | SRR13214148 | SLE | Blood | NA | NA |
| PRJNA683207 | SRR13214149 | SLE | Blood | NA | NA |
| PRJNA683207 | SRR13214150 | SLE | Blood | NA | NA |
| PRJNA684605 | SRR13243280 | SLE | Blood | NA | NA |
| PRJNA684605 | SRR13243281 | SLE | Blood | NA | NA |
| PRJNA684605 | SRR13243282 | SLE | Blood | NA | NA |
| PRJNA684605 | SRR13243283 | SLE | Blood | NA | NA |
| PRJNA684605 | SRR13243284 | SLE | Blood | NA | NA |
| PRJNA684605 | SRR13243285 | SLE | Blood | NA | NA |
| PRJNA684605 | SRR13243286 | Control | Blood | NA | NA |
| PRJNA684605 | SRR13243287 | Control | Blood | NA | NA |
| PRJNA684605 | SRR13243288 | Control | Blood | NA | NA |
| PRJNA684605 | SRR13243289 | Control | Blood | NA | NA |
| PRJNA684605 | SRR13243290 | Control | Blood | NA | NA |
| PRJNA684605 | SRR13243291 | Control | Blood | NA | NA |
| PRJNA684605 | SRR13243292 | Control | Blood | NA | NA |
| PRJNA684605 | SRR13243293 | SLE | Blood | NA | NA |
| PRJNA684605 | SRR13243294 | SLE | Blood | NA | NA |
| PRJNA684605 | SRR13243295 | SLE | Blood | NA | NA |
| PRJNA684605 | SRR13243296 | SLE | Blood | NA | NA |
| PRJNA684605 | SRR13243297 | SLE | Blood | NA | NA |
| PRJNA684605 | SRR13243298 | SLE | Blood | NA | NA |
| PRJNA684605 | SRR13243299 | SLE | Blood | NA | NA |
| PRJNA684605 | SRR13243300 | SLE | Blood | NA | NA |
| PRJNA684605 | SRR13243301 | SLE | Blood | NA | NA |
| PRJNA684605 | SRR13243302 | SLE | Blood | NA | NA |
| PRJNA684605 | SRR13243303 | SLE | Blood | NA | NA |
| PRJNA684605 | SRR13243304 | SLE | Blood | NA | NA |
| PRJNA684605 | SRR13243305 | SLE | Blood | NA | NA |
| PRJNA684605 | SRR13243306 | SLE | Blood | NA | NA |
| PRJNA715175 | SRR13988787 | Control | Blood | NA | NA |
| PRJNA715175 | SRR13988788 | SLE | Blood | NA | NA |
| PRJNA715175 | SRR13988789 | SLE | Blood | NA | NA |
| PRJNA715175 | SRR13988790 | SLE | Blood | NA | NA |
| PRJNA715175 | SRR13988791 | SLE | Blood | NA | NA |
| PRJNA715175 | SRR13988792 | SLE | Blood | NA | NA |
| PRJNA715175 | SRR13988793 | SLE | Blood | NA | NA |
| PRJNA715175 | SRR13988794 | SLE | Blood | NA | NA |
| PRJNA715175 | SRR13988795 | Control | Blood | NA | NA |
| PRJNA715175 | SRR13988796 | Control | Blood | NA | NA |
| PRJNA715175 | SRR13988797 | Control | Blood | NA | NA |
| PRJNA715175 | SRR13988798 | Control | Blood | NA | NA |
| PRJNA715175 | SRR13988799 | Control | Blood | NA | NA |
| PRJNA717024 | SRR14063573 | SLE | Blood | 26 | female |
| PRJNA717024 | SRR14063574 | SLE | Blood | 27 | female |
| PRJNA717024 | SRR14063575 | SLE | Blood | 60 | female |
| PRJNA717024 | SRR14063576 | SLE | Blood | 24 | male |
| PRJNA717024 | SRR14063577 | SLE | Blood | 44 | female |
| PRJNA717024 | SRR14063578 | SLE | Blood | 19 | female |
| PRJNA717024 | SRR14063579 | SLE | Blood | 51 | female |
| PRJNA717024 | SRR14063580 | SLE | Blood | 50 | female |
| PRJNA717024 | SRR14063581 | SLE | Blood | 23 | female |
| PRJNA717024 | SRR14063582 | SLE | Blood | 52 | female |
| PRJNA717024 | SRR14063583 | Control | Blood | 26 | NA |
| PRJNA717024 | SRR14063584 | SLE | Blood | 52 | female |
| PRJNA717024 | SRR14063585 | SLE | Blood | 35 | female |
| PRJNA717024 | SRR14063586 | SLE | Blood | 25 | female |
| PRJNA717024 | SRR14063587 | SLE | Blood | 58 | female |
| PRJNA717024 | SRR14063588 | SLE | Blood | 54 | female |
| PRJNA717024 | SRR14063589 | SLE | Blood | 20 | female |
| PRJNA717024 | SRR14063590 | SLE | Blood | 63 | female |
| PRJNA717024 | SRR14063591 | SLE | Blood | 37 | female |
| PRJNA717024 | SRR14063592 | SLE | Blood | 51 | female |
| PRJNA717024 | SRR14063593 | SLE | Blood | 19 | female |
| PRJNA717024 | SRR14063594 | Control | Blood | 53 | NA |
| PRJNA717024 | SRR14063595 | SLE | Blood | 19 | female |
| PRJNA717024 | SRR14063596 | SLE | Blood | 40 | female |
| PRJNA717024 | SRR14063597 | SLE | Blood | 39 | female |
| PRJNA717024 | SRR14063598 | SLE | Blood | 22 | female |
| PRJNA717024 | SRR14063599 | SLE | Blood | 24 | female |
| PRJNA717024 | SRR14063600 | SLE | Blood | 46 | female |
| PRJNA717024 | SRR14063601 | SLE | Blood | 30 | female |
| PRJNA717024 | SRR14063602 | SLE | Blood | 30 | female |
| PRJNA717024 | SRR14063603 | SLE | Blood | 37 | female |
| PRJNA717024 | SRR14063604 | SLE | Blood | 54 | female |
| PRJNA717024 | SRR14063605 | Control | Blood | 50 | NA |
| PRJNA717024 | SRR14063606 | SLE | Blood | 54 | female |
| PRJNA717024 | SRR14063607 | SLE | Blood | 37 | female |
| PRJNA717024 | SRR14063608 | SLE | Blood | 52 | female |
| PRJNA717024 | SRR14063609 | SLE | Blood | 46 | female |
| PRJNA717024 | SRR14063610 | SLE | Blood | 47 | female |
| PRJNA717024 | SRR14063611 | SLE | Blood | 63 | female |
| PRJNA717024 | SRR14063612 | SLE | Blood | 46 | female |
| PRJNA717024 | SRR14063613 | SLE | Blood | 43 | female |
| PRJNA717024 | SRR14063614 | SLE | Blood | 33 | female |
| PRJNA717024 | SRR14063615 | SLE | Blood | 28 | male |
| PRJNA717024 | SRR14063616 | Control | Blood | 49 | NA |
| PRJNA717024 | SRR14063617 | SLE | Blood | 34 | female |
| PRJNA717024 | SRR14063618 | SLE | Blood | 33 | male |
| PRJNA717024 | SRR14063619 | SLE | Blood | 56 | female |
| PRJNA717024 | SRR14063620 | SLE | Blood | 24 | female |
| PRJNA717024 | SRR14063621 | SLE | Blood | 43 | female |
| PRJNA717024 | SRR14063622 | SLE | Blood | 35 | male |
| PRJNA717024 | SRR14063623 | SLE | Blood | 29 | female |
| PRJNA717024 | SRR14063624 | SLE | Blood | 22 | female |
| PRJNA717024 | SRR14063625 | SLE | Blood | 27 | female |
| PRJNA717024 | SRR14063626 | SLE | Blood | 27 | female |
| PRJNA717024 | SRR14063627 | Control | Blood | 32 | NA |
| PRJNA717024 | SRR14063628 | SLE | Blood | 34 | female |
| PRJNA717024 | SRR14063629 | SLE | Blood | 43 | female |
| PRJNA717024 | SRR14063630 | Control | Blood | 35 | female |
| PRJNA717024 | SRR14063631 | Control | Blood | 40 | female |
| PRJNA717024 | SRR14063632 | Control | Blood | 36 | female |
| PRJNA717024 | SRR14063633 | Control | Blood | 51 | female |
| PRJNA717024 | SRR14063634 | Control | Blood | 35 | female |
| PRJNA717024 | SRR14063635 | Control | Blood | 38 | female |
| PRJNA717024 | SRR14063636 | Control | Blood | 57 | female |
| PRJNA717024 | SRR14063637 | Control | Blood | 37 | female |
| PRJNA717024 | SRR14063638 | Control | Blood | 39 | female |
| PRJNA717024 | SRR14063639 | Control | Blood | 36 | female |
| PRJNA717024 | SRR14063640 | Control | Blood | 54 | female |
| PRJNA717024 | SRR14063641 | Control | Blood | 39 | female |
| PRJNA717024 | SRR14063642 | Control | Blood | 51 | female |
| PRJNA717024 | SRR14063643 | Control | Blood | 55 | female |
| PRJNA717024 | SRR14063644 | Control | Blood | 53 | female |
| PRJNA717024 | SRR14063645 | Control | Blood | 46 | female |
| PRJNA717024 | SRR14063646 | Control | Blood | 60 | female |
| PRJNA717024 | SRR14063647 | Control | Blood | 40 | female |
| PRJNA717024 | SRR14063648 | Control | Blood | 29 | male |
| PRJNA717024 | SRR14063649 | Control | Blood | 34 | female |
| PRJNA717024 | SRR14063650 | Control | Blood | 29 | male |
| PRJNA717024 | SRR14063651 | Control | Blood | 21 | female |
| PRJNA717024 | SRR14063652 | Control | Blood | 24 | NA |
| PRJNA717024 | SRR14063653 | Control | Blood | 22 | NA |
| PRJNA717024 | SRR14063654 | Control | Blood | 20 | female |
| PRJNA717024 | SRR14063655 | Control | Blood | 22 | female |
| PRJNA717024 | SRR14063656 | Control | Blood | 27 | NA |
| PRJNA717024 | SRR14063657 | Control | Blood | 33 | NA |
| PRJNA717024 | SRR14063658 | Control | Blood | 28 | NA |
| PRJNA717024 | SRR14063659 | Control | Blood | 20 | NA |
| PRJNA717024 | SRR14063660 | Control | Blood | 33 | NA |
| PRJNA717024 | SRR14063661 | Control | Blood | 59 | female |
| PRJNA717024 | SRR14063662 | Control | Blood | 28 | female |
| PRJNA717024 | SRR14063663 | Control | Blood | 24 | male |
| PRJNA717024 | SRR14063664 | Control | Blood | 30 | female |
| PRJNA717024 | SRR14063665 | Control | Blood | 23 | NA |
| PRJNA717024 | SRR14063666 | Control | Blood | 33 | NA |
| PRJNA717024 | SRR14063667 | Control | Blood | 54 | NA |
| PRJNA717024 | SRR14063668 | Control | Blood | 42 | NA |
| PRJNA717024 | SRR14063669 | Control | Blood | 25 | NA |
| PRJNA717024 | SRR14063670 | Control | Blood | 51 | female |
| PRJNA717024 | SRR14063671 | Control | Blood | 41 | female |
| PRJNA717024 | SRR14063672 | Control | Blood | 47 | NA |
| PRJNA734019 | SRR14694911 | SLE | Blood | NA | NA |
| PRJNA734019 | SRR14694912 | SLE | Blood | NA | NA |
| PRJNA734019 | SRR14694913 | SLE | Blood | NA | NA |
| PRJNA734019 | SRR14694914 | SLE | Blood | NA | NA |
| PRJNA734019 | SRR14694915 | SLE | Blood | NA | NA |
| PRJNA734019 | SRR14694916 | Control | Blood | NA | NA |
| PRJNA734019 | SRR14694917 | Control | Blood | NA | NA |
| PRJNA734019 | SRR14694918 | Control | Blood | NA | NA |
| PRJNA734019 | SRR14694919 | Control | Blood | NA | NA |
| PRJNA734019 | SRR14694920 | Control | Blood | NA | NA |
| PRJNA734293 | SRR14701392 | Control | Blood | NA | NA |
| PRJNA734293 | SRR14701393 | Control | Blood | NA | NA |
| PRJNA734293 | SRR14701394 | Control | Blood | NA | NA |
| PRJNA734293 | SRR14701395 | Control | Blood | NA | NA |
| PRJNA734293 | SRR14701396 | Control | Blood | NA | NA |
| PRJNA734293 | SRR14701397 | Control | Blood | NA | NA |
| PRJNA734293 | SRR14701398 | Control | Blood | NA | NA |
| PRJNA734293 | SRR14701399 | SLE | Blood | NA | NA |
| PRJNA734293 | SRR14701400 | SLE | Blood | NA | NA |
| PRJNA734293 | SRR14701401 | SLE | Blood | NA | NA |
| PRJNA734293 | SRR14701402 | SLE | Blood | NA | NA |
| PRJNA734293 | SRR14701403 | SLE | Blood | NA | NA |
| PRJNA734293 | SRR14701404 | Control | Blood | NA | NA |
| PRJNA734293 | SRR14701405 | SLE | Blood | NA | NA |
| PRJNA734293 | SRR14701406 | SLE | Blood | NA | NA |
| PRJNA734293 | SRR14701407 | SLE | Blood | NA | NA |
| PRJNA734293 | SRR14701408 | SLE | Blood | NA | NA |
| PRJNA734293 | SRR14701409 | SLE | Blood | NA | NA |
| PRJNA734293 | SRR14701410 | SLE | Blood | NA | NA |
| PRJNA734293 | SRR14701411 | SLE | Blood | NA | NA |
| PRJNA734293 | SRR14701412 | SLE | Blood | NA | NA |
| PRJNA734293 | SRR14701413 | Control | Blood | NA | NA |
| PRJNA734293 | SRR14701414 | Control | Blood | NA | NA |
| PRJNA734293 | SRR14701415 | Control | Blood | NA | NA |
| PRJNA734293 | SRR14701416 | Control | Blood | NA | NA |
| PRJNA734293 | SRR14701417 | Control | Blood | NA | NA |
| PRJNA734293 | SRR14701418 | Control | Blood | NA | NA |
| PRJNA734293 | SRR14701419 | Control | Blood | NA | NA |
| PRJNA734293 | SRR14701420 | SLE | Blood | NA | NA |
| PRJNA734293 | SRR14701421 | Control | Blood | NA | NA |
| PRJNA734293 | SRR14701422 | SLE | Blood | NA | NA |
| PRJNA734293 | SRR14701423 | Control | Blood | NA | NA |
| PRJNA734293 | SRR14701424 | SLE | Blood | NA | NA |
| PRJNA734293 | SRR14701425 | SLE | Blood | NA | NA |
| PRJNA734293 | SRR14701426 | SLE | Blood | NA | NA |
| PRJNA734293 | SRR14701427 | SLE | Blood | NA | NA |
| PRJNA734293 | SRR14701428 | SLE | Blood | NA | NA |
| PRJNA734293 | SRR14701429 | SLE | Blood | NA | NA |
| PRJNA734293 | SRR14701430 | SLE | Blood | NA | NA |
| PRJNA858861 | SRR20214023 | SLE | Blood | 41 years | female |
| PRJNA858861 | SRR20214024 | SLE | Blood | 58 years | female |
| PRJNA858861 | SRR20214025 | SLE | Blood | 27 years | male |
| PRJNA858861 | SRR20214026 | SLE | Blood | 38 years | female |
| PRJNA858861 | SRR20214027 | SLE | Blood | 30 years | female |
| PRJNA858861 | SRR20214028 | SLE | Blood | 27 years | female |
| PRJNA858861 | SRR20214029 | SLE | Blood | 33 years | female |
| PRJNA858861 | SRR20214030 | SLE | Blood | 44 years | female |
| PRJNA858861 | SRR20214031 | SLE | Blood | 37 years | female |
| PRJNA858861 | SRR20214032 | SLE | Blood | 46 years | female |
| PRJNA858861 | SRR20214033 | SLE | Blood | 55 years | female |
| PRJNA858861 | SRR20214034 | SLE | Blood | 47 years | female |
| PRJNA858861 | SRR20214035 | SLE | Blood | 30 years | female |
| PRJNA858861 | SRR20214036 | SLE | Blood | 62 years | female |
| PRJNA858861 | SRR20214037 | SLE | Blood | 43 years | female |
| PRJNA858861 | SRR20214038 | SLE | Blood | 41 years | female |
| PRJNA858861 | SRR20214039 | SLE | Blood | 58 years | female |
| PRJNA858861 | SRR20214040 | SLE | Blood | 27 years | male |
| PRJNA858861 | SRR20214041 | SLE | Blood | 38 years | female |
| PRJNA858861 | SRR20214042 | SLE | Blood | 30 years | female |
| PRJNA858861 | SRR20214043 | SLE | Blood | 27 years | female |
| PRJNA858861 | SRR20214044 | SLE | Blood | 30 years | female |
| PRJNA858861 | SRR20214045 | SLE | Blood | 32 years | female |
| PRJNA858861 | SRR20214046 | SLE | Blood | 32 years | female |
| PRJNA858861 | SRR20214047 | SLE | Blood | 49 years | female |
| PRJNA858861 | SRR20214048 | SLE | Blood | 47 years | female |
| PRJNA858861 | SRR20214049 | SLE | Blood | 33 years | female |
| PRJNA858861 | SRR20214050 | SLE | Blood | 44 years | female |
| PRJNA858861 | SRR20214051 | SLE | Blood | 37 years | female |
| PRJNA858861 | SRR20214052 | SLE | Blood | 46 years | female |
| PRJNA858861 | SRR20214053 | SLE | Blood | 55 years | female |
| PRJNA858861 | SRR20214054 | SLE | Blood | 47 years | female |
| PRJNA858861 | SRR20214055 | SLE | Blood | 62 years | female |
| PRJNA858861 | SRR20214056 | SLE | Blood | 43 years | female |
| PRJNA858861 | SRR20214057 | SLE | Blood | 49 years | female |
| PRJNA858861 | SRR20214058 | SLE | Blood | 47 years | female |

**Supplementary Table S2.** The virus abundances detected in each Run.

|  |  |  |  |  |  |  |  |  |  |  |
| --- | --- | --- | --- | --- | --- | --- | --- | --- | --- | --- |
| **Run** | **HHV3** | **HHV4** | **HHV5** | **TTV** | **AV-sp** | **HAdV-C** | **PGV-A** | **PGV-C** | **IAV** | **EMCV** |
| SRR10342368 | 0.00 | 0.00 | 0.06 | 0.00 | 0.00 | 0.00 | 0.00 | 0.00 | 0.00 | 0.00 |
| SRR10342369 | 0.00 | 0.00 | 8.24 | 5.08 | 7.45 | 0.00 | 0.00 | 0.00 | 0.00 | 13.68 |
| SRR12190424 | 0.00 | 0.00 | 0.62 | 0.00 | 0.00 | 0.00 | 0.00 | 0.00 | 0.00 | 0.00 |
| SRR12190436 | 0.00 | 0.00 | 0.10 | 0.00 | 0.00 | 0.00 | 0.00 | 0.00 | 0.00 | 0.00 |
| SRR12190437 | 0.00 | 0.00 | 0.27 | 0.00 | 0.00 | 0.00 | 0.00 | 0.00 | 0.00 | 0.00 |
| SRR13214142 | 0.00 | 0.06 | 0.00 | 0.00 | 0.00 | 0.00 | 0.00 | 0.00 | 0.00 | 0.00 |
| SRR14063580 | 0.00 | 0.00 | 0.00 | 0.00 | 0.00 | 0.00 | 5.47 | 4.46 | 0.00 | 0.00 |
| SRR14063598 | 0.00 | 0.03 | 0.00 | 0.00 | 0.00 | 0.00 | 0.00 | 0.00 | 0.00 | 0.00 |
| SRR14063599 | 0.00 | 0.00 | 0.00 | 2.18 | 0.00 | 0.00 | 0.00 | 0.00 | 0.00 | 0.00 |
| SRR14063601 | 0.00 | 0.00 | 0.23 | 0.00 | 0.00 | 0.00 | 0.00 | 0.00 | 0.00 | 0.00 |
| SRR14063612 | 0.00 | 0.00 | 0.00 | 0.00 | 0.00 | 0.00 | 183.36 | 81.58 | 0.00 | 0.00 |
| SRR14063617 | 0.00 | 0.03 | 0.00 | 0.00 | 0.00 | 0.00 | 0.00 | 0.00 | 0.00 | 0.00 |
| SRR14063618 | 0.00 | 0.03 | 0.00 | 0.00 | 0.00 | 0.00 | 0.00 | 0.00 | 0.00 | 0.00 |
| SRR14063619 | 0.00 | 0.00 | 0.00 | 0.00 | 2.42 | 0.00 | 0.00 | 0.00 | 0.00 | 0.00 |
| SRR14063622 | 0.00 | 0.00 | 0.00 | 0.00 | 0.00 | 0.00 | 1.91 | 2.00 | 0.00 | 0.00 |
| SRR14063626 | 0.00 | 0.05 | 0.00 | 0.00 | 0.00 | 0.00 | 0.00 | 0.00 | 0.00 | 0.00 |
| SRR14063640 | 0.00 | 0.09 | 0.00 | 0.00 | 0.00 | 0.00 | 0.00 | 0.00 | 0.00 | 0.00 |
| SRR14063644 | 0.00 | 0.00 | 0.00 | 0.00 | 3.13 | 0.00 | 0.00 | 0.00 | 0.00 | 0.00 |
| SRR14063650 | 0.00 | 0.00 | 0.00 | 0.00 | 0.00 | 0.00 | 4.36 | 0.00 | 0.00 | 0.00 |
| SRR14063659 | 0.00 | 0.00 | 0.00 | 0.00 | 0.00 | 0.00 | 81.86 | 82.02 | 0.00 | 0.00 |
| SRR14063660 | 0.00 | 0.07 | 0.00 | 0.00 | 0.00 | 0.00 | 0.00 | 0.00 | 0.00 | 0.00 |
| SRR14694912 | 0.00 | 1.16 | 0.00 | 0.00 | 0.00 | 0.00 | 0.00 | 0.00 | 0.00 | 0.00 |
| SRR14694914 | 0.00 | 0.11 | 0.00 | 0.00 | 0.00 | 0.00 | 0.00 | 0.00 | 0.00 | 0.00 |
| SRR20214031 | 0.00 | 0.13 | 0.00 | 0.00 | 0.00 | 0.00 | 0.00 | 0.00 | 0.00 | 0.00 |
| SRR20214045 | 0.00 | 0.00 | 0.00 | 0.00 | 9.66 | 0.00 | 0.00 | 0.00 | 0.00 | 0.00 |
| SRR20214046 | 0.00 | 0.00 | 0.00 | 0.00 | 9.71 | 0.00 | 0.00 | 0.00 | 0.00 | 0.00 |
| SRR3362713 | 0.00 | 0.00 | 0.06 | 0.00 | 0.00 | 0.80 | 0.00 | 0.00 | 0.00 | 0.00 |
| SRR3362715 | 0.00 | 0.25 | 0.00 | 0.00 | 0.00 | 0.00 | 0.00 | 0.00 | 0.00 | 0.00 |
| SRR3362723 | 0.00 | 0.00 | 0.00 | 0.00 | 5.67 | 0.00 | 0.00 | 0.00 | 0.00 | 0.00 |
| SRR3362725 | 0.00 | 0.00 | 0.17 | 0.00 | 0.00 | 0.00 | 0.00 | 0.00 | 0.00 | 0.00 |
| SRR3362729 | 0.00 | 0.00 | 0.06 | 0.00 | 0.00 | 0.00 | 0.00 | 0.00 | 0.00 | 0.00 |
| SRR3362733 | 0.00 | 0.25 | 0.00 | 0.00 | 0.00 | 0.00 | 0.00 | 0.00 | 0.00 | 0.00 |
| SRR3362734 | 0.00 | 0.00 | 0.00 | 3.53 | 0.00 | 0.00 | 0.00 | 0.00 | 0.00 | 0.00 |
| SRR7367609 | 0.00 | 0.00 | 0.00 | 0.00 | 0.00 | 0.00 | 0.00 | 0.00 | 0.65 | 0.00 |
| SRR7367625 | 0.00 | 0.00 | 0.00 | 0.00 | 0.00 | 0.64 | 0.00 | 0.00 | 0.00 | 0.00 |
| SRR7367644 | 0.00 | 0.00 | 0.07 | 0.00 | 0.00 | 0.00 | 0.00 | 0.00 | 0.00 | 0.00 |
| SRR7367651 | 0.00 | 0.00 | 0.07 | 0.00 | 0.00 | 0.00 | 0.00 | 0.00 | 0.00 | 0.00 |
| SRR7367660 | 0.00 | 0.00 | 0.00 | 2.05 | 0.00 | 0.00 | 0.00 | 0.00 | 0.00 | 0.00 |
| SRR7367661 | 0.00 | 0.00 | 0.03 | 0.00 | 3.01 | 0.00 | 0.00 | 0.00 | 0.00 | 0.00 |
| SRR7367664 | 0.00 | 0.00 | 0.03 | 4.07 | 11.93 | 0.00 | 0.00 | 0.00 | 0.00 | 0.00 |
| SRR7367672 | 0.00 | 0.05 | 0.00 | 0.00 | 0.00 | 0.43 | 0.00 | 0.00 | 0.00 | 0.00 |
| SRR7367675 | 0.00 | 0.00 | 0.00 | 0.00 | 0.00 | 0.22 | 0.00 | 0.00 | 0.00 | 0.00 |
| SRR7367684 | 0.00 | 0.00 | 0.00 | 2.00 | 0.00 | 0.00 | 0.00 | 0.00 | 0.00 | 0.00 |
| SRR7367695 | 0.00 | 0.00 | 0.23 | 0.00 | 0.00 | 0.00 | 0.00 | 0.00 | 0.00 | 0.00 |
| SRR7367701 | 0.00 | 0.00 | 0.13 | 0.00 | 0.00 | 0.00 | 0.00 | 0.00 | 0.00 | 0.00 |
| SRR7367703 | 0.00 | 0.00 | 0.00 | 0.00 | 0.00 | 0.21 | 0.00 | 0.00 | 0.00 | 0.00 |
| SRR7367706 | 0.00 | 0.00 | 0.00 | 0.00 | 3.01 | 0.00 | 0.00 | 0.00 | 0.00 | 0.00 |
| SRR7367716 | 0.00 | 0.00 | 0.13 | 0.00 | 0.00 | 0.00 | 0.00 | 0.00 | 0.00 | 0.00 |
| SRR7367735 | 0.00 | 0.00 | 0.00 | 6.07 | 0.00 | 0.00 | 0.00 | 0.00 | 0.00 | 0.00 |
| SRR7367742 | 0.00 | 0.00 | 0.00 | 3.34 | 0.00 | 0.00 | 0.00 | 0.00 | 0.00 | 0.00 |
| SRR7367754 | 0.00 | 0.00 | 0.00 | 0.00 | 0.00 | 0.00 | 0.00 | 0.00 | 3.56 | 0.00 |
| SRR7367763 | 0.00 | 0.00 | 0.03 | 0.00 | 0.00 | 0.00 | 0.00 | 0.00 | 0.00 | 0.00 |
| SRR7367771 | 0.00 | 0.00 | 0.00 | 2.06 | 0.00 | 0.00 | 0.00 | 0.00 | 0.00 | 0.00 |
| SRR7367790 | 0.00 | 0.00 | 0.00 | 2.04 | 0.00 | 0.00 | 0.00 | 0.00 | 0.00 | 0.00 |
| SRR7367795 | 0.00 | 0.00 | 0.00 | 0.00 | 0.00 | 0.00 | 0.00 | 0.00 | 1.17 | 0.00 |
| SRR7367802 | 0.00 | 0.04 | 0.00 | 0.00 | 0.00 | 0.00 | 0.00 | 0.00 | 0.00 | 0.00 |
| SRR7367809 | 0.00 | 0.00 | 2.38 | 6.16 | 3.01 | 0.00 | 0.00 | 0.00 | 0.00 | 0.00 |
| SRR7367868 | 0.00 | 0.00 | 0.00 | 0.00 | 0.00 | 0.21 | 0.00 | 0.00 | 0.00 | 0.00 |
| SRR7367875 | 0.00 | 0.04 | 0.00 | 0.00 | 0.00 | 0.00 | 0.00 | 0.00 | 0.00 | 0.00 |
| SRR7367876 | 0.00 | 0.04 | 0.00 | 0.00 | 0.00 | 0.00 | 0.00 | 0.00 | 0.00 | 0.00 |
| SRR7367880 | 0.00 | 0.00 | 0.03 | 0.00 | 0.00 | 0.00 | 0.00 | 0.00 | 0.00 | 0.00 |
| SRR7367892 | 0.00 | 0.00 | 0.10 | 0.00 | 0.00 | 0.00 | 0.00 | 0.00 | 0.00 | 0.00 |
| SRR7367902 | 0.00 | 0.00 | 0.00 | 0.00 | 0.00 | 0.00 | 0.00 | 0.00 | 1.18 | 0.00 |
| SRR7367919 | 0.00 | 0.00 | 0.00 | 8.30 | 30.42 | 0.00 | 0.00 | 0.00 | 3.53 | 0.00 |
| SRR7367923 | 0.00 | 0.00 | 0.10 | 0.00 | 0.00 | 0.00 | 0.00 | 0.00 | 0.00 | 0.00 |
| SRR7367938 | 0.00 | 0.00 | 0.00 | 6.15 | 18.02 | 0.00 | 0.00 | 0.00 | 0.00 | 0.00 |
| SRR7367941 | 0.00 | 0.00 | 0.00 | 0.00 | 6.04 | 0.00 | 0.00 | 0.00 | 0.00 | 0.00 |
| SRR7367943 | 0.00 | 0.00 | 0.07 | 0.00 | 0.00 | 0.00 | 0.00 | 0.00 | 0.00 | 0.00 |
| SRR7367944 | 0.00 | 0.04 | 0.00 | 0.00 | 0.00 | 0.00 | 0.00 | 0.00 | 0.00 | 0.00 |
| SRR7367948 | 0.00 | 0.00 | 0.00 | 0.00 | 0.00 | 0.24 | 0.00 | 0.00 | 0.00 | 0.00 |
| SRR7367979 | 0.00 | 0.00 | 0.00 | 0.00 | 2.95 | 0.00 | 0.00 | 0.00 | 0.00 | 0.00 |
| SRR7367988 | 0.00 | 0.00 | 0.03 | 0.00 | 0.00 | 0.00 | 0.00 | 0.00 | 0.00 | 0.00 |
| SRR7367991 | 0.18 | 0.00 | 0.00 | 0.00 | 0.00 | 0.00 | 0.00 | 0.00 | 0.00 | 0.00 |
| SRR7367993 | 0.00 | 0.00 | 0.00 | 0.00 | 9.14 | 0.00 | 0.00 | 0.00 | 0.00 | 0.00 |
| SRR7368000 | 0.00 | 0.04 | 0.00 | 0.00 | 0.00 | 0.00 | 0.00 | 0.00 | 0.00 | 0.00 |
| SRR7368002 | 0.06 | 0.00 | 0.00 | 0.00 | 0.00 | 0.00 | 0.00 | 0.00 | 0.00 | 0.00 |
| SRR7368012 | 0.00 | 0.00 | 0.00 | 2.12 | 3.11 | 0.00 | 0.00 | 0.00 | 0.00 | 0.00 |
| SRR7368017 | 0.00 | 0.00 | 0.00 | 0.00 | 2.98 | 0.00 | 0.00 | 0.00 | 0.00 | 0.00 |
| SRR7368032 | 0.00 | 0.00 | 0.00 | 0.00 | 0.00 | 0.21 | 0.00 | 0.00 | 0.00 | 0.00 |
| SRR7368050 | 0.00 | 0.00 | 0.03 | 0.00 | 0.00 | 0.00 | 0.00 | 0.00 | 0.00 | 0.00 |
| SRR7368055 | 0.00 | 0.00 | 0.22 | 0.00 | 0.00 | 0.00 | 0.00 | 0.00 | 0.00 | 0.00 |
| SRR7368056 | 0.00 | 0.00 | 0.00 | 0.00 | 0.00 | 0.74 | 0.00 | 0.00 | 0.00 | 0.00 |
| SRR7368063 | 0.00 | 0.00 | 0.05 | 0.00 | 0.00 | 0.00 | 0.00 | 0.00 | 0.00 | 0.00 |
| SRR8074220 | 0.00 | 0.40 | 0.03 | 0.00 | 0.00 | 0.00 | 0.00 | 0.00 | 0.00 | 0.00 |
| SRR8074222 | 0.00 | 0.00 | 0.00 | 12.44 | 0.00 | 0.00 | 0.00 | 0.00 | 0.00 | 0.00 |
| SRR8074223 | 0.00 | 0.00 | 0.05 | 3.03 | 0.00 | 0.00 | 0.00 | 0.00 | 0.00 | 0.00 |
| SRR8181364 | 0.00 | 0.08 | 0.00 | 0.00 | 0.00 | 0.00 | 0.00 | 0.00 | 0.00 | 0.00 |
| SRR8181373 | 0.00 | 0.00 | 0.00 | 0.00 | 0.00 | 1.22 | 0.00 | 0.00 | 0.00 | 0.00 |
| SRR8181375 | 0.00 | 0.00 | 0.00 | 0.00 | 0.00 | 0.93 | 0.00 | 0.00 | 0.00 | 0.00 |
| SRR8181376 | 0.00 | 0.00 | 0.00 | 0.00 | 0.00 | 0.36 | 0.00 | 0.00 | 0.00 | 0.00 |
| SRR8181377 | 0.00 | 0.00 | 0.00 | 0.00 | 0.00 | 0.53 | 0.00 | 0.00 | 0.00 | 0.00 |

| **Supplementary Table S3**. Results of Logistic Regression Analysis for the Impact of Age and Sex on Virus Positivity and SLE Status | | | | | | |
| --- | --- | --- | --- | --- | --- | --- |
| **Variable** | **Coefficient** | **Standard** | **Error** | **z-value** | **P>\|z\|** | **95% Confidence Interval** |
| Virus | Age | -0.0850 | 0.385 | -0.221 | 0.825 | [-0.840, 0.670] |
| SLE | Age | 0.4026 | 0.454 | 0.887 | 0.375 | [-0.487, 1.292] |
| Virus | Sex | -1.204 | 0.658 | -1.829 | 0.067 | [-2.494, 0.086] |
| SLE | Sex | -0.1542 | 0.556 | -0.277 | 0.782 | [-1.245, 0.936] |

**Supplementary Table S4.** The taxonomic details about the viruses identified in the study.

**Supplementary Table S5.** The ISGs used in the study and their categories.

|  |  |  |  |  |  |
| --- | --- | --- | --- | --- | --- |
| **ISG name** | **Antiviral** | **SLE-related** | **ISG name** | **Antiviral** | **SLE-related** |
| RSAD2 | 1 | 1 | MYD88 | 1 | 0 |
| OAS3 | 1 | 1 | N4BP1 | 1 | 0 |
| IFIT3 | 1 | 1 | NOD1 | 1 | 0 |
| PARP9 | 1 | 1 | NUB1 | 1 | 0 |
| CMPK2 | 1 | 1 | PARP10 | 1 | 0 |
| USP18 | 1 | 1 | PARP12 | 1 | 0 |
| DDX60 | 1 | 1 | PARP14 | 1 | 0 |
| OAS2 | 1 | 1 | PHF11 | 1 | 0 |
| EPSTI1 | 1 | 1 | PLAC8 | 1 | 0 |
| DDX58 | 1 | 1 | PSMB8 | 1 | 0 |
| IFIT1 | 1 | 1 | PSMB9 | 1 | 0 |
| MX1 | 1 | 1 | PSME1 | 1 | 0 |
| IFIT2 | 1 | 1 | PSME2 | 1 | 0 |
| IFI44 | 1 | 1 | RAB24 | 1 | 0 |
| IFIH1 | 1 | 1 | RNF114 | 1 | 0 |
| RTP4 | 1 | 1 | RNF213 | 1 | 0 |
| IFI35 | 1 | 1 | SAMD9L | 1 | 0 |
| ZBP1 | 1 | 1 | SAMHD1 | 1 | 0 |
| IFITM3 | 1 | 1 | SAT1 | 1 | 0 |
| IRF7 | 1 | 1 | SCARB2 | 1 | 0 |
| LGALS3BP | 1 | 1 | SERPINB9 | 1 | 0 |
| ISG15 | 1 | 1 | SLFN5 | 1 | 0 |
| LY6E | 1 | 1 | SP110 | 1 | 0 |
| SPATS2L | 1 | 1 | SP140 | 1 | 0 |
| SIGLEC1 | 1 | 1 | STAMBPL1 | 1 | 0 |
| ABTB2 | 1 | 0 | STAT1 | 1 | 0 |
| ACOT9 | 1 | 0 | STAT2 | 1 | 0 |
| ADAR | 1 | 0 | STAT3 | 1 | 0 |
| AGRN | 1 | 0 | TAP1 | 1 | 0 |
| ANKFY1 | 1 | 0 | TDRD7 | 1 | 0 |
| ATP10A | 1 | 0 | TMEM106A | 1 | 0 |
| B2M | 1 | 0 | TMEM140 | 1 | 0 |
| BAG1 | 1 | 0 | TNFSF10 | 1 | 0 |
| BST2 | 1 | 0 | TRAFD1 | 1 | 0 |
| CASP1 | 1 | 0 | TRIM21 | 1 | 0 |
| CASP4 | 1 | 0 | TRIM25 | 1 | 0 |
| CHMP5 | 1 | 0 | UBE2L6 | 1 | 0 |
| CNP | 1 | 0 | UNC93B1 | 1 | 0 |
| DHX58 | 1 | 0 | VRK2 | 1 | 0 |
| DTX3L | 1 | 0 | XAF1 | 1 | 0 |
| EIF2AK2 | 1 | 0 | ZC3HAV1 | 1 | 0 |
| FAM26F | 1 | 0 | ZCCHC2 | 1 | 0 |
| FBXO39 | 1 | 0 | ZNFX1 | 1 | 0 |
| FBXO6 | 1 | 0 | IFI27 | 0 | 1 |
| GBP2 | 1 | 0 | IFI44L | 0 | 1 |
| GBP3 | 1 | 0 | OAS1 | 0 | 1 |
| GBP4 | 1 | 0 | IFI6 | 0 | 1 |
| GBP5 | 1 | 0 | CXCL10 | 0 | 1 |
| GBP6 | 1 | 0 | HERC5 | 0 | 1 |
| GSDMD | 1 | 0 | OASL | 0 | 1 |
| HPSE | 1 | 0 | SERPING1 | 0 | 1 |
| HSH2D | 1 | 0 | PDCD1LG2 | 0 | 1 |
| IFITM1 | 1 | 0 | PLSCR1 | 0 | 1 |
| IFITM2 | 1 | 0 | S100A8 | 0 | 1 |
| IRF1 | 1 | 0 | CCL2 | 0 | 1 |
| IRF9 | 1 | 0 | IFIT5 | 0 | 1 |
| ISG20 | 1 | 0 | FCGR1A | 0 | 1 |
| LAP3 | 1 | 0 | LAMP3 | 0 | 1 |
| LCP2 | 1 | 0 | HERC6 | 0 | 1 |
| LGALS9 | 1 | 0 | SOCS1 | 0 | 1 |
| LY96 | 1 | 0 | S100A9 | 0 | 1 |
| LYSMD2 | 1 | 0 | SOCS3 | 0 | 1 |
| MAP3K8 | 1 | 0 | MS4A4A | 0 | 1 |
| MLKL | 1 | 0 | C1QB | 0 | 1 |
| MX2 | 1 | 0 |  |  |  |
